# Translocation of green fluorescent protein in homo- and hetero-transgrafted plants

**DOI:** 10.5511/plantbiotechnology.24.0501b

**Published:** 2024-12-25

**Authors:** Takumi Ogawa, Kanae Kato, Harue Asuka, Yumi Sugioka, Tomofumi Mochizuki, Hirokazu Fukuda, Takumi Nishiuchi, Taira Miyahara, Hiroaki Kodama, Daisaku Ohta

**Affiliations:** 1 Graduate School of Agriculture, Osaka Metropolitan University, Naka-ku, Sakai, Osaka 599-8531, Japan; 2 Graduate School of Life and Environmental Sciences, Osaka Prefecture University, Naka-ku, Sakai, Osaka 599-8531, Japan; 3 Graduate School of Engineering, Osaka Metropolitan University, Naka-ku, Sakai, Osaka 599-8531, Japan; 4 Graduate School of Natural Science and Technology, Kanazawa University, Kakuma, Kanazawa, Ishikawa 920-1192, Japan; 5 Research Center for Experimental Modeling of Human Disease, Kanazawa University, Takaramachi, Kanazawa, Ishikawa 920-8640, Japan; 6 Graduate School of Horticulture, Chiba University, Inage-ku, Chiba, Chiba 263-8522, Japan

**Keywords:** green fluorescent protein (GFP), new plant breeding technologies (NPBTs), tobacco, tomato, transgrafting

## Abstract

Transgrafting, a technique involving the use of genetically modified (GM) plants as grafting partners with non-genetically modified (non-GM) crops, presents non-GM edible harvests from transgrafted crops, often considered as non-GM products. However, the classification of the non-GM portions from transgrafted crops as non-GM foods remains uncertain, therefore it is critical to investigate the potential translocation of substances from GM portions to non-GM edible portions in transgrafted plants. In this study, we explored the translocation of exogenous proteins (luciferase and green fluorescent protein) in model transgrafted plants consisting of GM plant rootstocks and non-GM tomato scions. Our results suggest that exogenous proteins accumulated in the stem tissues of non-GM tomato scions in all cases investigated. The levels and patterns of exogenous protein accumulation in the non-GM tomato stem tissues varied among the individual transgrafted plants and rootstock plant species used. However, exogenous proteins were not detected in the fruits, the edible part of the tomato, and in mature leaves in non-GM tomato scions under the current experimental conditions. Our results provide basic knowledge for understanding exogenous protein translocation in transgrafted plants.

## Introduction

Grafting has been practiced since ancient times as an asexual plant propagation method, using plant parts from different plant varieties and, in some cases, different species (Melnyk and Meyerowitz 2015). Therefore, in fruit tree breeding and vegetable seedling production, grafting is an extremely useful and simple technology for leveraging the beneficial properties of grafting partners such as resistance to biotic and abiotic stresses, conferred by different genomes of cultivated or wild plant species used (Albacete et al. 2015; Kyriacou et al. 2017; Nawaz et al. 2016). Furthermore, recent developments in molecular biology and genetic engineering could lead to the use of genetically modified (GM) plants for grafting. The grafting strategy using GM plants as the grafting partners with non-genetically modified (non-GM) crops is referred to as transgrafting (Eckerstorfer et al. 2019; Tsaballa et al. 2021). In addition to traditional genetic modification, various useful traits have been developed with the aid of new plant breeding techniques (NPBTs), particularly precise sequence manipulation using genome editing technology. Those newly developed plants from NPBTs will also be applied to the development of transgrafted crops (Eckerstorfer et al. 2019; Nawaz et al. 2016).

Implementation of transgrafting is expected to accelerate the development of agriculturally and commercially attractive crops applicable for cultivation under the current climate extremes. So far, however, grafting has not been a universal technology, in that it is applicable to plants of taxonomical proximity (Goldschmidt 2014; Notaguchi et al. 2020; Rasool et al. 2020). The mechanism of grafting incompatibility between different plant species has not been fully understood, and the elucidation of the molecular mechanisms underlying the successful formation of graft unions should contribute to interspecies grafting, making use of useful traits of different plant species (Loupit and Cookson 2020). Recently, it has been reported that transgenic overexpression of β-1,4-glucanase facilitates cell–cell adhesion at the graft unions thereby promoting interspecies transgrafting (Notaguchi et al. 2020). Removing the barrier of grafting incompatibility should accelerate the utilization of valuable traits, such as the tolerance toward biotic and abiotic stresses, better yields, and nutrient value, from not only wild species but also GM plants of different taxonomical origins.

Considering transgrafting, an obvious benefit is that the GM portions are physically separated from the non-GM edible parts. Therefore, the non-GM edible harvests from transgrafted crops may be regarded as non-GM products and, therefore, the lengthy and costly processes of authorization and registration may not be required. However, it has not been established whether the non-GM parts from transgrafted crops can be treated as non-GM foods. As a food safety concern, it should be assessed whether the qualities of the non-GM harvests may be different from those of the original non-GM crops due to possible influences, directly or indirectly, from the GM parts. In particular, it is critical to study the possible exchange of substances such as toxic metabolites and/or bioactive molecules between the GM portions and the non-GM edible portions of transgrafted plants. It is known that biological macromolecules such as proteins, mRNAs, small RNAs, and bioactive substances, including hormones, are transported across grafting sites (Kehr and Kragler 2018; Kyriacou et al. 2017; Tsaballa et al. 2021). Furthermore, it has also been demonstrated that the exchange of genome information per se occurs between neighboring cells around the graft junctions (Fuentes et al. 2014; Hertle et al. 2021; Tanwar et al. 2023; Wang et al. 2017). Therefore, the possibility of transgene transfer from the GM portion to the non-GM portion of transgrafted plants cannot be excluded. Furthermore, it has been reported that grafting induces genome-wide changes in DNA methylation profiles (Cerruti et al. 2021; Kapazoglou et al. 2021; Kodama et al. 2022; Tamiru et al. 2018; Wu et al. 2013), suggesting that epigenetic mechanisms could eventually influence the quality of the edible parts of transgrafted crops. These observations indicate that GM portions, even if they are physically separated from non-GM portions in transgrafted plants, could affect the quality of non-GM edible parts.

When interspecies grafting and transgrafting become universal and widely used in crop production, new concerns arise regarding the safety of using the harvests from such grafted plants as food. As described above, some substances are moving across the graft junction, supporting the growth of grafted plants. In other words, to dispel the concerns about food safety, the possible transfer of toxic substances and bioactive molecules, including transgene products, from grafting partners must be evaluated on a scientific basis. In a previous study, we reported the accumulation of nicotine, a toxic secondary metabolite, in the tomato fruits of hetero-grafted plants composed of tobacco rootstocks and tomato scions (Ogawa et al. 2023). More recently, Miyahara et al. reported that recombinant luciferase (Luc) protein was observed to transfer from GM portions to non-GM portions across the grafting unions in transgrafted tobacco plants (*Nicotiana tabacum* cv. SR1; Miyahara et al. 2024). This finding was unexpected as Luc is a well established reporter protein typically expected to remain localized within the expressing cell. However, it is possible that this observation may be an exceptional event specific to the combination of Luc protein and homo-transgrafted *N. tabacum* plants.

To explore this phenomenon further, we investigated the translocation of Luc protein in hetero-transgrafted plants composed of Luc-expressing *N. tabacum* rootstocks and non-GM tomato scions. Additionally, we also investigated the translocation of green fluorescent protein (GFP) from GFP-expressing rootstocks to non-GM tomato scions in the following three cases: (1) homo-transgrafted plants with GFP-expressing tomato rootstocks; (2) hetero-transgrafted plants with GFP-expressing *Nicotiana benthamiana* rootstocks; and (3) hetero-transgrafted plants with GFP-expressing *N. tabacum* rootstocks. In all investigated cases, the movement of exogenous proteins (GFP and Luc) was observed in the stem tissues but not in the mature leaves and fruits of non-GM tomato scions under our experimental conditions. Our results provide a basic knowledge of the exogenous protein translocation in transgrafted plants.

## Materials and methods

### Plant materials

Tomato (*Solanum lycopersicum* L. cv. Micro-Tom) and two *Nicotiana* species (*Nicotiana benthamiana*, *Nicotiana tabacum* L. cv. SR1) were used throughout this study. Seeds of these plants were surface sterilized, washed five times with sterile distilled water, and germinated on a half-strength Murashige and Skoog (MS) medium (Murashige and Skoog 1962) supplemented with 0.8% (w/v) agar, 1.5% (w/v) sucrose under a long-day photoperiod (16 h light/8 h dark) at 25°C. After two weeks, the seedlings were transplanted into plastic pot containing potting soil (Golden Granular Potting Soil; Iris Ohyama, Miyagi, Japan) and were grown under a long-day photoperiod (16 h light/8 h dark) at 25°C.

### Generation of GFP-expressing transgenic plants

To construct a plant expression vector for GFP expression, a DNA fragment (approximately 1.5 kbp) containing the Cauliflower mosaic virus 35S RNA (CaMV35S) promoter, sGFP (S65T) cDNA (Chiu et al. 1996), and nopaline synthase gene (NOS) terminator was inserted into the HindIII site and EcoRI site of a pBINPLUS vector (van Engelen et al. 1995), resulting in the construction of a pBINPLUS-35S:GFP vector. *Rhizobium radiobacter* strain GV3101 harboring this vector was cultured at 28°C with continuous shaking at 220 rpm for 18 h in 3 ml of LB medium containing 50 mg l^−1^ kanamycin. The bacterial cells were then collected by centrifugation at 6,000×g for 5 min and resuspended to an OD_600_ of 0.2 in *Agrobacterium* minimal medium (Chilton et al. 1974) containing 50 mg l^−1^ kanamycin and 200 µM acetosyringone. Subsequently, the bacterial cell suspension (10 ml) was cultured at 28°C with continuous shaking at 220 rpm for 18 h. After centrifugation at 6,000×g for 5 min, the bacterial cells were resuspended to an OD_600_ of 0.01 in MS medium containing 0.003% (v/v) 2-mercaptoethanol and 200 µM acetosyringone. This bacterial cell suspension was used as an infection medium for plant transformation.

*Agrobacterium*-mediated plant transformation was performed according to the method described by Hashimoto et al. (2018). Shortly, cotyledons from five-day-old tomato seedlings and true leaves from three-week-old tobacco plants (*N. benthamiana*, *N. tabacum*) were utilized. These plant leaves were cut into small pieces (tomato: 5–7 mm in length; tobacco: 5 mm square) using a sterile razor blade. The explant was immersed in the infection medium for 10 min with orbital shaking and then transferred to a co-cultivation medium [MS medium with 0.8% (w/v) agar, 3% (w/v) sucrose, and 40 µM acetosyringone], ensuring that the adaxial surface was in contact with the medium. The explants were cultured in the dark at 25°C for 3 days. Subsequently, the explants were transferred to callus-inducing medium [MS medium with 0.8% (w/v) agar, 3% (w/v) sucrose, 1.5 mg l^−1^ *trans*-zeatin, 100 mg l^−1^ kanamycin, and 25 mg l^−1^ meropenem], placing the abaxial surface in contact with the medium. Cultivation continued under a long-day photoperiod (16 h light/8 h dark) at 25°C. After two weeks, the explant was moved to fresh medium. At four weeks after transformation, the obtained kanamycin-resistant calluses were transferred to shoot-inducing medium [MS medium with 0.8% (w/v) agar, 3% (w/v) sucrose, 1.0 mg l^−1^ *trans*-zeatin, 100 mg l^−1^ kanamycin, and 25 mg l^−1^ meropenem] and cultivation continued for several weeks. The kanamycin-resistant shoots, which emerged from the calluses, were excised using a sterile razor blade and transferred to root-inducing medium [half-strength MS medium with 0.8% (w/v) agar, 1.5% (w/v) sucrose, 50 mg l^−1^ kanamycin, and 25 mg l^−1^ meropenem]. Following root development, the regenerated plant was transplanted into soil and maintained under high humidity conditions for the initial 10 days and then grown under normal growth conditions. These regenerated plants were used for genotyping and fluorescence observation to confirm the presence of transgene and expression of GFP.

### Genotyping of regenerated plants

Total DNA was extracted from the leaf samples of regenerated plants using the MagExtractor Plant Genome kit (TOYOBO, Osaka, Japan) according to the manufacturer’s protocol. The DNA concentration of the extract was estimated by measuring the absorbance at 260 nm in a BioSpec-nano spectrophotometer (Shimadzu, Kyoto, Japan). To confirm whether the regenerated plants retained the transgene (Supplementary Figure S1A), the DNA fragment corresponding to the *GFP* region was amplified by PCR method using the TaKaRa Ex Taq Hot Start version (Takara Bio, Shiga, Japan) and oligo-DNA primers (Fasmac, Kanagawa, Japan) shown in Supplementary Table S1. PCR was performed in a final volume of 10 µl mixture containing 0.2 U Ex *Taq* DNA polymerase HS, 1× Ex *Taq* buffer (Mg^2+^ plus), 0.2 mM of each dNTP, 1 µM forward primer, 1 µM reverse primer, and 10 ng total DNA extracted from samples. The PCR program was set as follows: initial denaturation at 94°C for 2 min; 30 cycles of 94°C for 30 s, 60°C for 30 s, and 72°C for 30 s; followed by final extension at 72°C for 30 s. For positive control reactions, the tomato α-*Tubulin* gene (NCBI accession number: XM_004244485) and the predicted *N. tabacum* *Actin7* gene (NCBI accession number: XM_016658880) were amplified in the same reaction using specific primers (Supplementary Table S1).

### Plant grafting

Grafting was performed by a splice-grafting method. To adjust the diameter of the stems, four-week-old tomato and *N. benthamiana* plants and seven-week-old *N. tabacum* plants were used for grafting. The grafting combination is shown in Table 1. The stems of plants used for grafting were cut diagonally approximately 2 cm above the soil surface using a sterile razor blade. The cut surface of two plants was aligned and fixed by a graft tube with an inner diameter of 2.3 mm (Nasunics Co. Ltd.) and graft clip (Japan Peerless Industries Co. Ltd.). After grafting, plants were grown under a long-day photoperiod (16 h light/8 h dark) at 25°C in a plant cultivation room. Axillary buds were removed from each of the grafted plants once a week during the growth using a sterile razor blade.

**Table 1:** Table 1. A list of grafted plants used in this study.

Grafted plant groups (rootstock/scion)	Types of grafting	Rootstock		Scion plant#	Number of grafted plants	References
		Plant	Transgene			
Nt/MT	Heterograft	non-GM N. tabacum***	—	non-GM tomato	5	Ogawa et al. (2023)
NtLuc/MT	Hetero-transgraft	GM *N. tabacum*	*Luc* gene	do.	5	do.
MT/MT	Homograft	non-GM tomato*	—	do.	3	in this study
MTGFP/MT	Homo-transgraft	GM tomato	*GFP* gene	do.	3	do.
Nb/MT	Heterograft	non-GM N. benthamiana**	—	do.	4	do.
NbGFP/MT	Hetero-transgraft	GM *N. benthamiana*	*GFP* gene	do.	2	do.
Nt/MT	Heterograft	non-GM N. tabacum***	—	do.	3	do.
NtGFP/MT	Hetero-transgraft	GM *N. tabacum*	*GFP* gene	do.	3	do.

# All grafted plants were produced using non-GM tomato scion. * *Solanum lycopersicum* L. cv. Micro-Tom. ** *Nicotiana benthamiana* L. *** *Nicotiana tabacum* L. cv. SR1. do., same as above.

### Fluorescence observation

The tomato fruits were harvested from the grafted plants 10 days after breaker (DAB) between 11 and 16 weeks after grafting. From each fruit, the exocarp (outer skin) tissues were excised and used for fluorescence observation. Stem and leaf samples were prepared from each grafted plant 16 weeks after grafting. For leaves, mature leaves were sampled from the bottom, middle, and upper portions of the scion on each grafted plant. Leaf disks of 2 mm in diameter were excised from each leaf sample. These leaf disks were fixed in a solution containing 4% paraformaldehyde in phosphate-buffered saline (PBS; 137 mM NaCl, 8.1 mM Na_2_HPO_4_, 2.68 mM KCl, 1.47 mM KH_2_PO_4_, pH 7.6) under reduced pressure conditions (−0.08 MPa) for 4 min. Following fixation, the samples were rinsed with PBS before being subjected to fluorescence observation. As for the remaining stem tissue, it was cut horizontally at 10 mm below and above the graft junction to obtain the stem section, including the graft junction. The remaining stem tissue was cut horizontally at 10-mm intervals from the bottom cut surface to create a series of stem sections at various distances from the graft junction. The longitudinal sections, approximately 0.2 mm in thickness, were excised from each stem section and subjected to fixation using the same 4% paraformaldehyde solution as the leaf samples and used for fluorescence observation. All plant tissues were mounted onto glass slides using PBS as the mounting medium and observed using a confocal laser scanning microscope (LSM700; Carl Zeiss, Germany). For GFP excitation, a 488-nm laser was used, and the emission wavelength was set within the range 490–520 nm to eliminate interference from chlorophyll autofluorescence (Okazawa et al. 2022).

### Semi-quantitative RT-PCR

To assess transcript accumulation levels, we used a semi-quantitative reverse transcription (RT)-PCR method. Tissues were collected from grafted plants 16 weeks after grafting, rapidly frozen in liquid nitrogen and stored at −80°C until further analysis. Total RNA was extracted from these samples using the ISOSPIN Plant RNA kit (NIPPON GENE, Tokyo, Japan), following the manufacturer’s instructions. The concentrations of RNA in the extracted samples were determined by measuring the absorbance at 260 nm in a BioSpec-nano spectrophotometer (Shimadzu). cDNA was synthesized from the total RNA using the PrimeScript RT-PCR Kit (Takara Bio), following the manufacturer’s protocol. For conversion of mRNA into first-strand cDNA through reverse transcription, an oligo(dT) primer was used. The cDNA corresponding to the transgene was amplified by PCR using the TaKaRa *Ex Taq* Hot Start version (Takara Bio) and oligo-DNA primers (Fasmac) as detailed in Supplementary Table S1. In addition of the target gene, the tomato α-*Tubulin* gene (NCBI accession number: XM_004244485) and the predicted *N. tabacum* *Actin7* gene (NCBI accession number: XM_016658880) were used as reference genes for RT-PCR analysis.

### In vivo luciferase imaging

To conduct in vivo Luc imaging, the root of the grafted plant was washed with distilled water and subsequently immersed in a 0.4 mM luciferin solution for 2 h at room temperature. Following this incubation, the whole plant was sprayed with a 1.0 mM luciferin solution. Immediately after that, the plant was placed in a light-tight chamber to prevent external light interference. Photons emitted during the luciferase reaction within the plant were captured and recorded using a cooled charge-coupled device (CCD) imaging system (C4742-80; Hamamatsu Photonics, Shizuoka, Japan).

### Measurement of luciferase activity using a luminometer

Luciferase assay was performed using the Luciferase Assay System (Promega, Tokyo, Japan) in accordance with the manufacturer’s protocol. The 10-DAB fruits were harvested from the grafted plants between 11 and 16 weeks after grafting. For preparation of leaf and stem tissues, grafted plants were used at 22 weeks after grafting. All samples were ground to a fine powder with a mortar and a pestle in liquid nitrogen. Proteins were extracted from the ground samples using cell culture lysis reagent [0.1 M phosphate buffer (pH 7.8), 1 mM EDTA, 7 mM 2-mercaptoethanol, 1% (w/v) Triton X-100, 10% (w/v) glycerol]. The resulting lysate was centrifuged at 12,000 rpm for 10 min at 4°C, and the supernatant was collected for the luciferase assay. Luminescence was measured using a luminometer (GENE Light 55; Microtec, Chiba, Japan) in a relative light units (RLU) measurement mode for a duration of 10 s at 25°C. The XL-Bradford kit (Pharma Foods International, Tokushima, Japan) was used to determine the protein concentration within the lysate. Absorbance measurement was taken at 595 nm using a UV-visible spectrophotometer (UV-2600; Shimadzu). Bovine serum albumin was used to make the calibration curve for the Bradford protein assay. The luciferase activities were represented as RLU per µg of protein in this study.

### Tissue printing and western blot analysis

Tissue printing and western blot analysis were performed according to the method described by Sunpapao et al. (2009) with slight modifications. Stem samples were prepared from each grafted plant 16 weeks after grafting. After removing all leaves from the main stem, the stem was cut horizontally 10 mm below the graft junction. Next, the stem tissue was cut horizontally 10 mm above the graft junction, yielding a stem section that included the graft junction. The remaining stem tissue was then cut horizontally at 10 mm intervals starting from the bottom cut surface, creating a series of stem sections varying distances from the graft junction. These stem sections were sliced to obtain longitudinal sections of 0.5 mm in thickness. After the cutting process, the longitudinal sections were placed onto a polyvinylidene difluoride (PVDF) membrane (ClearBlot P plus; ATTO, Tokyo, Japan) with the cut surface facing downward. The lid of the semi-dry blotting system (ATTO) was closed, and pressure was applied for 30 s. Before this step, the PVDF membrane was soaked in 99% methanol for 20 s, followed by immersion in Towbin buffer (Towbin et al. 1979) [25 mM Tris, 192 mM glycine, pH 8.3, 20% methanol] for 15 min at 22°C with gentle agitation. The membrane was then blocked by incubating in 5% skimmed milk for 30 min at room temperature. Then, the membrane was incubated with an anti-GFP mouse monoclonal antibody (cat. no. A-11120, Thermo Fisher Scientific) at a 1 : 1000 dilution in 1% skimmed milk for 16 h at 4°C. After washing the membrane with 1% skimmed milk for 15 min at room temperature twice, the membrane was incubated with an alkaline phosphatase-conjugated anti-mouse IgG(H+L) goat secondary antibody (Proteintech) at a 1 : 1000 dilution in 1% skimmed milk for 1 h at room temperature. The membrane was washed again with 1% skimmed milk for 15 min at room temperature, followed by additional washing with Tris-buffered saline with Tween 20 [50 mM Tris-HCl (pH 7.6), 150 mM NaCl, 0.05% (w/v) Tween 20, pH 7.6] for 15 min at room temperature. For the colorimetric detection of alkaline phosphatase activity, the membrane was incubated with the 5-bromo-4-chloro-3-indolyl-phosphate/nitro blue tetrazolium (BCIP/NBT) color development substrate (FUJIFILM Wako Pure Chemical) for 10–20 min at room temperature.

### Transcriptomic analysis of tomato fruits from hetero-grafted plants

Transcriptome analysis was performed as in previous papers (Ogawa et al. 2023). Briefly, total RNA was extracted from tomato fruits from each of the three Nb/MT and NbGFP/MT plants and the analysis was outsourced to the mRNA-seq service of Eurofin Genomics (Tokyo, Japan). The read data (BioProject ID: PRJDB15279, RUN ID: DRR504706–DRR504711) generated by NovaSeq6000 were filtered using fastp version 0.23.4 to remove adapter sequences. The read data were then aligned by rsem version 1.3.3 and bowtie2 version 2.5.1 to tomato cDNA sequence data (ITAG4.1_cDNA.fasta) and tobacco transcriptome data (Niben261_genome.annotation.transcripts.fasta) to obtain the expression level of each transcript. The GFP (GenBank accession: M62654) sequence was also checked for a corresponding sequence in the read data. Differentially expressed genes (DEGs) between the two groups were obtained from the expression data of each sample using the *edgeR* package version 3.40.2 in R version 4.3.1. PANTHER version 18.0 of the Gene Ontology (GO) resource (https://geneontology.org/ (Accessed Sep 29, 2023)) was used for GO analysis of the DEGs (Thomas et al. 2022).

### Proteomic analysis of tomato fruits from hetero-grafted plants

A shotgun proteomic analysis of proteins from tomato fruit was performed in the same manner as in previous studies (Ogawa et al. 2023). Three individuals each of NbGFP/MT and Nb/MT fruits were used for the analysis. The detected peptide sequences were annotated with the tomato (ITAG4.0_protein.fasta) and *N. benthamiana* (Niben261.peptide.fasta) protein data from the database site (https://solgenomics.net/ (Accessed Sep 25, 2023)). The proteins were considered as the differentially expressed proteins (DEPs) between groups if the detected peptide sequence matched two or more different regions of the attributed protein and the significant difference in expression levels between groups was *p*<0.05. Furthermore, if the peptide sequence differed from the tomato protein by more than two amino acids and was tobacco specific, the protein was considered to be from the tobacco rootstock. GO analysis of the DEPs was performed in the same manner as that of the transcriptomic analysis described above.

### Metabolic profiling

Non-targeted metabolic profiling of tomato fruits was performed following previously established methods (Ogawa et al. 2023). Liquid chromatography-electrospray ionization-mass spectrometry (LC-ESI-MS) was performed using the LCMS-8040 system with control through LabSolutions software (Shimadzu Corp., Kyoto, Japan). Tandem mass spectra for ion peak ID N366 (retention time: 5.59 min; *m*/*z*: 593.30) were acquired using the LCMS-8040 system operating in product ion scan mode, with the following settings: precursor ion: *m*/*z* 593.30; collision energy: −35 V; scan range: *m*/*z* 100–600.

### Statistical analyses

Principal component analysis (PCA) and Student’s *t*-test were conducted using MetaboAnalyst version 5.0 (Pang et al. 2021), a web tool designed for metabolomics data analysis (https://www.metaboanalyst.ca/home.xhtml (Accessed Sep 26, 2023)). Auto-scaling was selected as the data standardization method for PCA.

## Results and discussion

### Translocation of luciferase in hetero-transgraft plants

Miyahara et al. (2024) reported the translocation of Luc protein across graft junctions, both from rootstock to scion and *vice versa*, in homo-transgraft plants composed of non-GM and Luc-expressing *N. tabacum*. To further investigate this observation in grafted plants with different combinations of plant species, we utilized hetero-transgraft plants composed of non-GM tomato (MT) scions and Luc-expressing *N. tabacum* (NtLuc) rootstocks (abbreviated to NtLuc/MT; Table 1). In prior multi-omics profiling of MT scion fruits harvested from the NtLuc/MT plants (Ogawa et al. 2023), transcriptome and proteome analyses did not detect Luc gene products (mRNA and protein), suggesting the absence of Luc protein in the MT scion fruits. Nevertheless, the translocation of Luc protein in MT scion leaves and stems of NtLuc/MT plants remains unexplored. To assess the translocation of Luc protein in the hetero-transgrafted NtLuc/MT plants, we utilized both in planta Luc imaging and luciferase assay with a luminometer, aligning the detection method with that of Miyahara et al. (2024).

To assess the translocation of Luc protein in NtLuc/MT plants, we first performed *in planta* Luc imaging. The roots of NtLuc/MT and its control Nt/MT plants were immersed in 0.4 mM luciferin solution for 2 h and the whole plants were sprayed with 1.0 mM luciferin solution just before the bioluminescence measurement. In control Nt/MT plants, no bioluminescence signal was observed from whole plants. In NtLuc/MT plants, bioluminescence signals were restrictedly observed from the NtLuc rootstock portions (root and stem) but not observed from the MT scion stem, leaf and fruits (Supplementary Figure S1). Two independent NtLuc/MT plants were observed and resulted in the same result. These results suggest that Luc protein had not accumulated in the MT scion tissues of NtLuc/MT plants, at least in cell layers near the plant surface.

**Figure 1:**
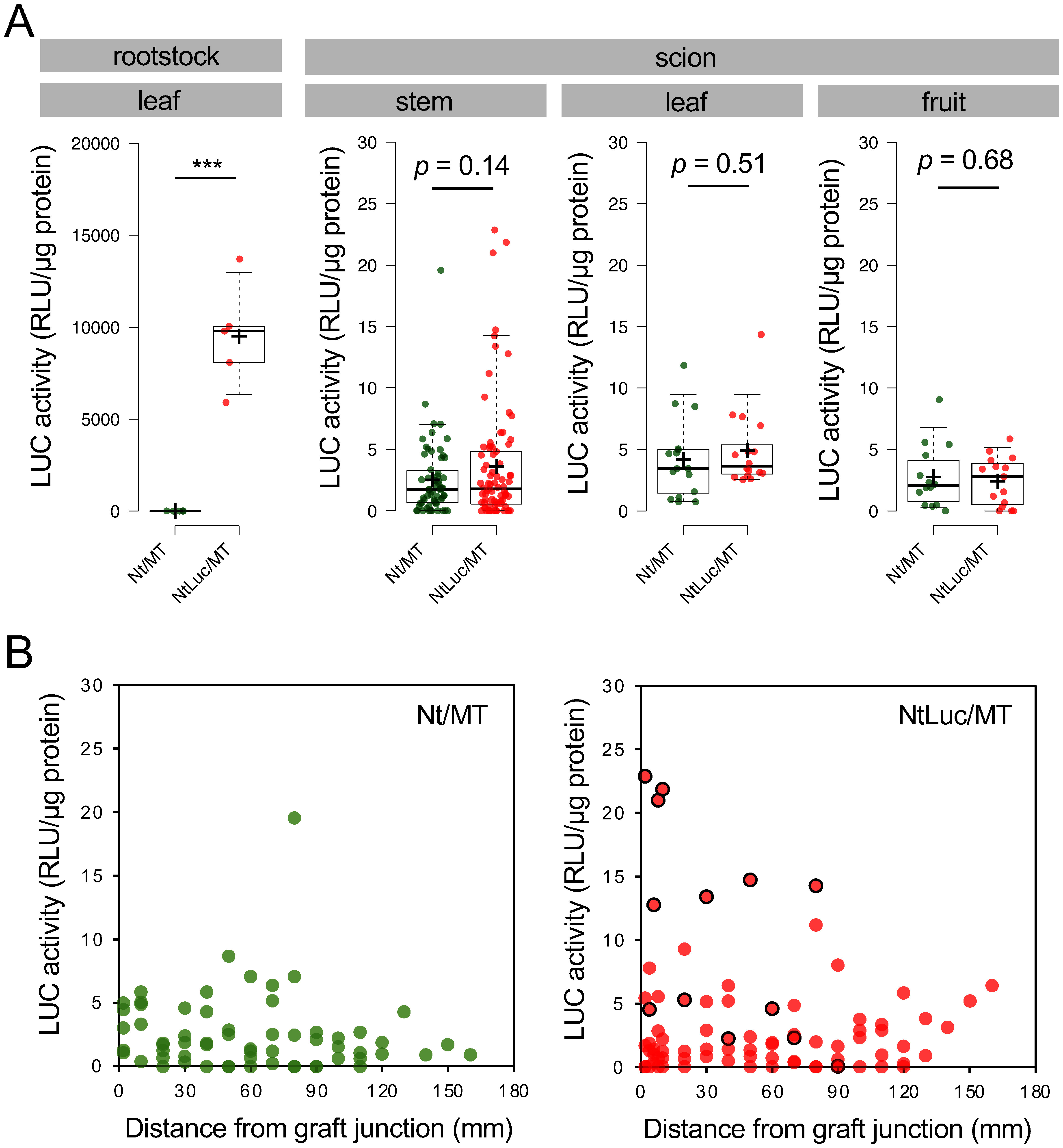
Figure 1. Luc activities of tissues from NtLuc/MT plants. (A) Luc activities of rootstock leaf, MT scion stems, leaves, and fruits were shown. (B) Luc activities of MT stem samples from Nt/MT and NtLuc/MT plants were plotted against the distance from the graft junction. Data from the NtLuc/MT #1 plant are circled in black.

Next, we measured Luc activity in tissue extracts prepared from NtLuc/MT plants using a luminometer. Regarding the Luc assay, the limit of detection (LOD) values were calculated using the 3σ method based on measurements of samples prepared from MT scion portions of Nt/MT plants. The LOD values for MT scion fruit, leaf, and stem were 10.4, 13.8, and 13.8 RLU/µg protein, respectively. There were no statistically significant differences (*p*<0.05) in Luc activities in MT scion stems, fruits and leaves between control Nt/MT plants and Nt/LucMT plants (Figure 1). Notably, Luc activities in several stem samples of NtLuc/MT plants were slightly higher than the LOD value for the MT scion stem (13.8 RLU/µg protein). We identified the source of these analytes to be from a NtLuc/MT plant (#1). We investigated the relationship between the observed Luc activity and distance from the graft junction in NtLuc/MT plant (#1), but no clear correlation was found (Figure 1). Luc mRNA was not detected from MT scion stem tissue 12–14 mm, 32–34 mm, and 82–84 mm apart from the graft junction in NtLuc/MT plants by RT-PCR (Supplementary Figure S2). These results indicated that Luc protein could be translocated to MT scion stem tissues in NtLuc/MT plants. However, similar to the observation in Miyahara et al. (2024), the rootstock-derived Luc protein was not always detected from MT scion stems and was not evenly distributed in stem tissues.

### Generation of model transgrafted plants composed of non-GM tomato scions and GFP-expressing plant rootstocks

To assess the translocation of GFP in transgrafted plants, we first generated transgenic tomato, *N. benthamiana*, and *N. tabacum* plants expressing GFP under the control of CaMV35S promoter (Supplementary Figure S3). The presence of a transgene in the regenerated plants was checked by PCR using a set of *GFP*-specific primers. As a result, three independent transgenic lines were obtained from each of the plant species (Supplementary Figure S3B). Based on the confocal microscopy analysis of GFP expression levels in leaf epidermal cells, transgenic tomato line #3, transgenic *N. benthamiana* line #2, and transgenic *N. tabacum* line #1 were selected as high GFP expression lines (Supplementary Figure S3C). These lines were self-pollinated, and T_3_-generation transgenic tomato plants (abbreviated as MTGFP), T_2_-generation transgenic *N. benthamiana* plants (abbreviated as NbGFP), and T_1_-generation transgenic *N. tabacum* plants (abbreviated as NtGFP) were used for further grafting experiments.

**Figure 2:**
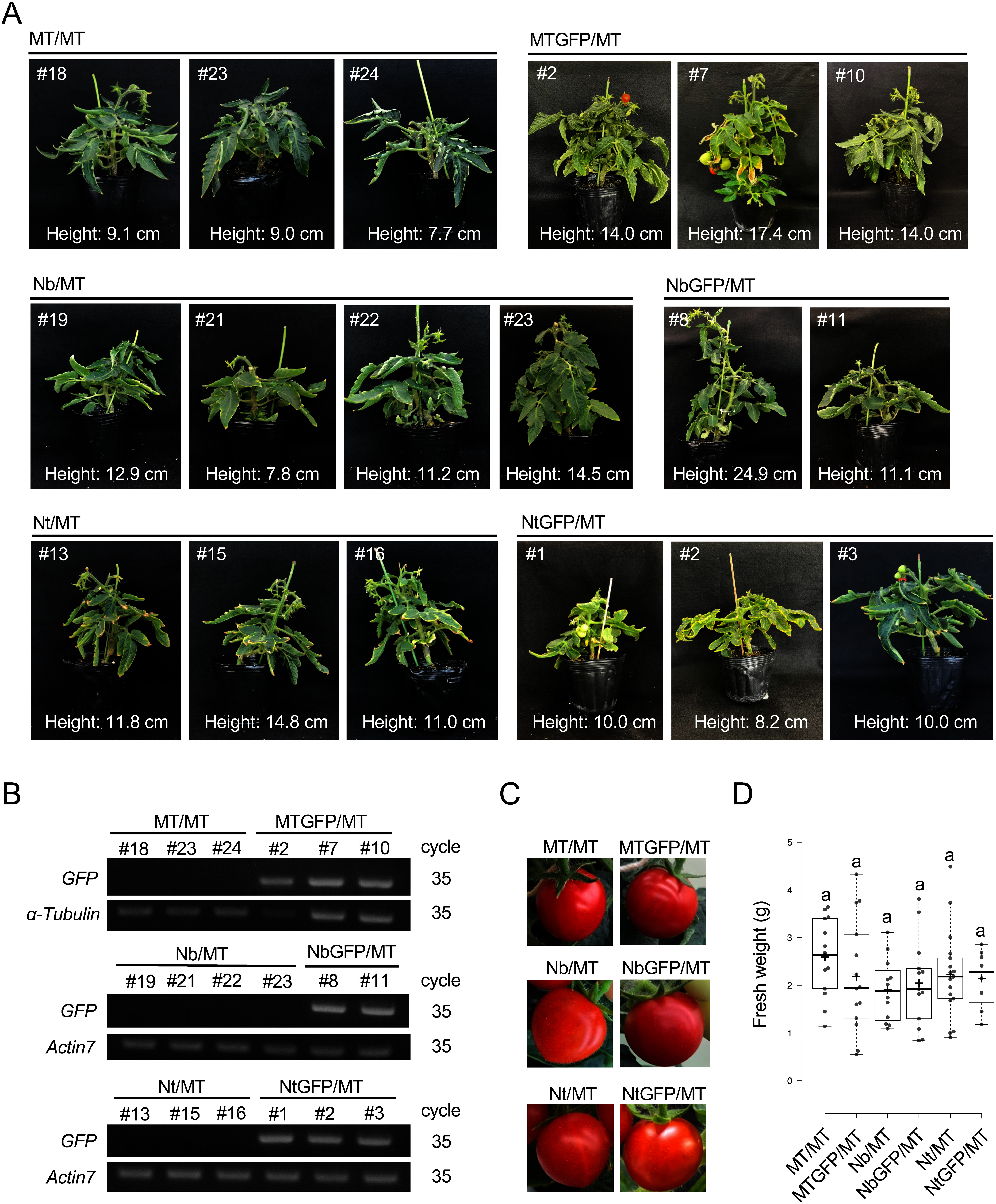
Figure 2. Grafted plants with GFP-expressing rootstocks used in this study. (A) Appearance of grafted plants. Photographs were taken at 16 weeks after grafting. (B) Semi-quantitative RT-PCR analysis of transgene expression in the rootstock portions of the grafted plants. The housekeeping genes, α-*Tubulin* and *Actin7*, were used as reference genes. (C) Appearance of MT scion fruits harvested from the grafted plants. Photographs were taken at 10 days after breaker. (D) Comparison of fresh weight on MT scion fruits from each of the grafted lines. Different letters indicate a significant difference (*p*<0.05) as determined by Tukey’s honestly significant difference (HSD) test.

We established three transgrafted plant groups composed of non-GM tomato (MT) and GFP-expressing plants (Table 1, Figure 2). The MT scions were grafted on MTGFP, NbGFP, and NtGFP to create a homo-transgraft line (MTGFP/MT) and two hetero-transgraft lines (NbGFP/MT and NtGFP/MT). We also established control grafted plants: MT scions were grafted on MT, non-GM *N. benthamiana* (Nb), and non-GM *N. tabacum* (Nt) to generate a homograft line (MT/MT) and two heterograft lines (Nb/MT and Nt/MT) (Table 1, Figure 2A). The GFP mRNA levels in the rootstock portion were confirmed by semi-quantitative RT-PCR (Figure 2B). The appearance and average fresh weight of MT scion fruits were similar among the grafted plant groups (Figure 2C, D). These grafted plants were used for further analyses.

### Translocation of GFP in homo-transgraft and hetero-transgraft plants

To assess the translocation of GFP across the graft junction, we sampled aerial part tissues (fruits, leaves, and stems) from homo-transgraft line (MTGFP/MT) and hetero-transgraft lines (NbGFP/MT and NtGFP/MT) and observed them using a confocal laser scanning microscopy. The results are summarized in Table 2.

**Table 2:** Table 2. Summary of the experimental results on fluorescence observation in the tissues of the homo-transgraft and hetero-transgraft plants.

Tissue	MTGFP/MT			NbGFP/MT		NtGFP/MT			Related data
	#2	#7	#10	#8	#11	#1	#2	#3	
Fruit*	−	−	−	−	−	−	−	−	Supplementary Figure S4
Leaf**									
bottom (1st)	−	−	−	−	−	−	−	−	Supplementary Figures S5, S6, S7
middle (4–6th)	−	−	−	−	−	−	−	−	do.
upper (7–12th)	−	−	−	−	−	−	−	−	do.
Stem***									
rootstock	+++	+++	++	+++	+++	++	++	+++	Supplementary Figures S8, S9, S10
scion (graft junction)	+	+	+	+++	+	nd	nd	nd	do.
scion (10–20 mm)	−	−	−	−	−	−	−	−	do.
scion (40–50 mm)	−	−	−	−	−	−	−	−	do.

−, no signal; +, weak signals; ++, moderate signals; +++, strong signals; nd, not determined. * Number of fruits samles used are 1–7, 3–4, and 2–5 from MTGFP/MT, NbGFP/MT, and NtGFP/MT, respectively. ** Leaf position is difined as counting up from the graft junction. *** For scion stem samples, distances from the graft junction are shown in parentheses. do., same as above.

First, we examined the presence of GFP in MT scion fruits, the edible part of tomato. The inner surface cell layers of fruit exocarp (outer skin) tissues were observed using confocal laser scanning microscopy (Table 2; Supplementary Figure S4). In positive control samples (non-grafted MTGFP), bright green fluorescence signals were detected in the nucleus and cytoplasm. In contrast, no green fluorescence signals were detected from either homo-transgraft group (MTGFP/MT) or two hetero-transgraft groups (NbGFP/MT and NtGFP/MT). No amplification product corresponding to GFP mRNA was detected from MT scion fruits in either homo-transgraft group (MTGFP/MT) or two hetero-transgraft groups (NbGFP/MT and NtGFP/MT) by RT-PCR (data not shown). Furthermore, *GFP* gene products (mRNA and protein) were not detected from the transcriptome and proteome data obtained from MT scion fruits of NbGFP/MT plants (Supplementary Tables S2, S3, S4, S5).

Next, we examined the presence of GFP in mature leaves of MT scions. The upper, middle, and lower leaves of MT scions were sampled from grafted plants and were observed using confocal laser scanning microscopy (Table 2; Supplementary Figures S5, S6, S7). In positive control samples (non-grafted MTGFP), bright green fluorescence signals were detected in the nucleus and cytoplasm. In contrast, no green fluorescence signals were detected from MT scion leaves of MTGFP/MT, NbGFP/MT and NtGFP/MT plants (Supplementary Figures S5, S6, S7). No amplification product corresponding to GFP mRNA was detected in semi-quantitative RT-PCR analysis using cDNA prepared from MT scion leaves obtained from MTGFP/MT, NbGFP/MT and NtGFP/MT plants (data not shown).

Finally, we examined the presence of GFP in MT scion stems. Stem tissues were sampled from the rootstock, graft junction, and scion (10–20 mm and 40–50 mm away from the graft junction) of the grafted plants and were observed using confocal laser scanning microscopy (Table 2; Supplementary Figures S8, S9, S10). In the positive control samples (non-grafted MTGFP) and rootstock stem samples of MTGFP/MT, NbGFP/MT and NtGFP/MT, bright green fluorescence signals were observed in the nucleus and cytoplasm. In contrast, no green fluorescence signals were detected from MT scion stems (both 10–20 mm and 40–50 mm away from the graft junction) of MTGFP/MT, NbGFP/MT and NtGFP/MT plants. Conversely, green fluorescence signals were observed on the MT scion side of the graft junction in MTGFP/MT, and NbGFP/MT plants (Supplementary Figures S8, S9). In three MTGFP/MT plants (#2, #7, and #10) and NbGFP/MT #11 plants, weak green fluorescence signals were observed on the MT scion side of the graft junction. However, as similar weak green fluorescence signals were occasionally observed in the control MT/MT and Nb/MT plants, it was difficult to conclusively attribute these signals solely to GFP. In contrast, bright green fluorescence signals were observed on the MT scion side of the graft junction in one of the two NbGFP/MT plants (#8). This intense green fluorescence was not observed in control Nb/MT scions; these signals were to be derived from GFP. The GFP mRNA was not detected in the MT scion stem tissues located 10–20 mm away from the graft junction in MTGFP/MT and NbGFP/MT plants (Supplementary Figure S11).

**Figure 3:**
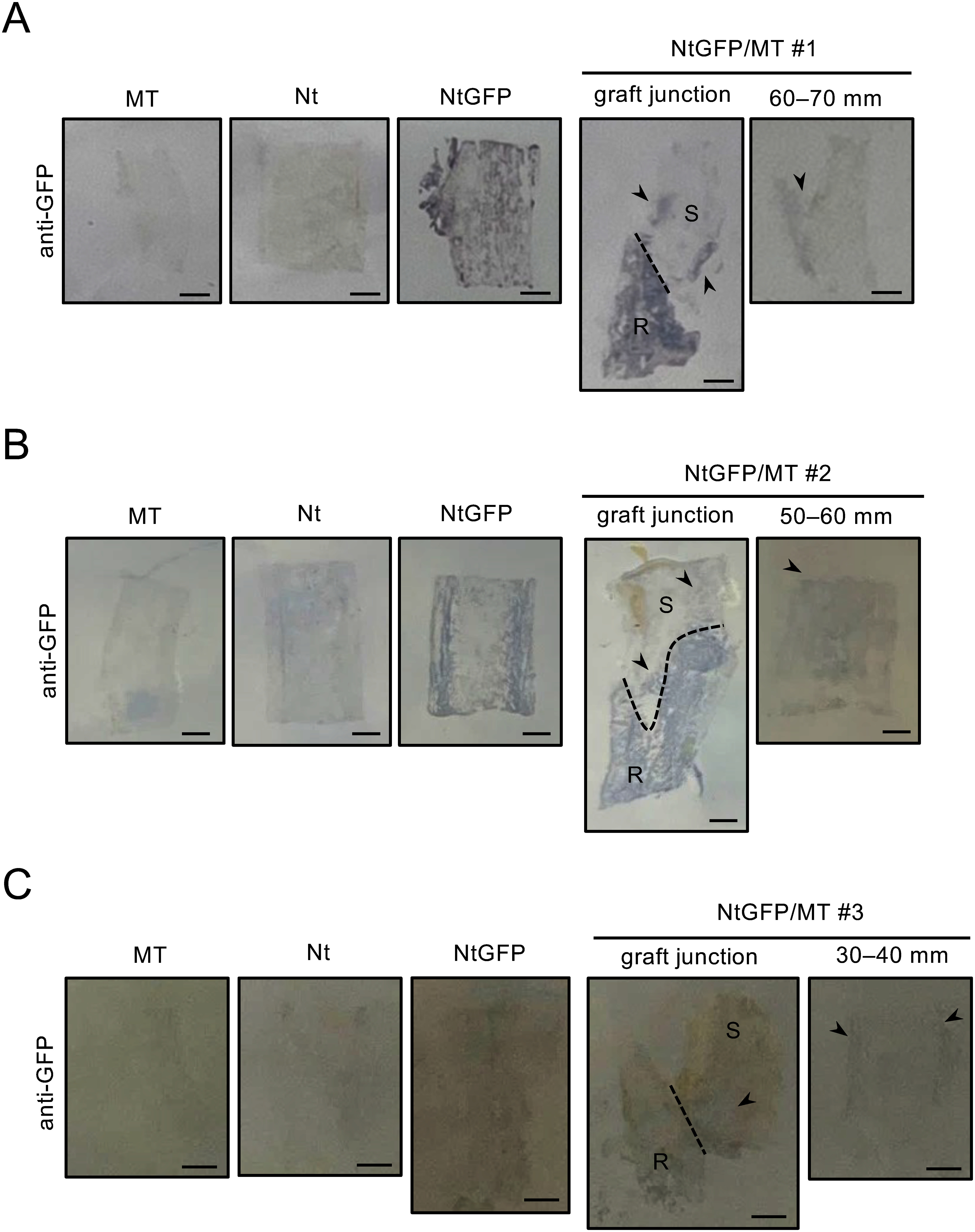
Figure 3. Tissue-print western blots of stem samples of NtGFP/MT plants. Stem sections of non-transgenic tomato (MT) and non-transgenic tobacco (Nt) were used as the negative control. Stem sections of GFP-expressing *N. tabacum* (NtGFP) were used as the positive control. Stem sections of the graft junction and MT scion portions of the NtGFP/MT #1 to #3 (A–C) were used for the analysis. For each plant, the positive and negative controls obtained on the same membrane are shown. R, rootstock; S, scion. The dashed line indicates the interface of the graft junction. The arrowheads indicate the GFP signals detected in the MT scion portions. Scale bars=2 mm.

In the control Nt/MT plants, intense green fluorescence signals were observed locally around the graft junction (Supplementary Figure S10). This suggests the accumulation of substance(s) with fluorescence properties similar to GFP around the graft junction when MT scions are grafted onto *N. tabacum* rootstocks. For this reason, we attempted to detect GFP in the MT stem tissues of NtGFP/MT plants by tissue printing and western blot analysis (Sunpapao et al. 2009). Stem sections of NtGFP/MT plants (#1–3) were blotted onto individual polyvinylidene difluoride membranes with negative (non-grafted MT and Nt) and positive (non-grafted NtGFP) control samples. Clear GFP signals were detected in the MT scion side of the graft junction in NtGFP/MT #1 plant, and very weak GFP signals were detected in the MT scion side of the graft junction in NtGFP/MT #2 and #3 plants. Additionally, weak GFP signals were detected locally on the MT scion stem sections in NtGFP/MT #1–3 plants (Figure 3). For example, in NtGFP/MT #1 plant, weak GFP signals were detected at the base region of the lateral branch 60–70 mm away from the graft junction (Figure 3). Semi-quantitative RT-PCR analysis was performed to detect GFP mRNA from the MT scion stem tissues of MTGFP/MT, NbGFP/MT, and NtGFP/MT. No GFP mRNA was detected in the MT scion stem tissues in all cases investigated (Supplementary Figure S11).

These results suggest that the transgene products of the *GFP* gene (mRNA and protein) were not translocated to MT scion fruits and mature leaves in MTGFP/MT, NbGFP/MT and NtGFP/MT plants, or the accumulation levels of the transgene products were below the detection limit in our experiments. In contrast, GFP could translocate from rootstocks to MT scion stem tissues near the graft junction, especially when *Nicotiana* plants are used as the rootstock. Furthermore, when using *N. tabacum* plants as rootstocks, rootstock-derived GFP could accumulate locally in MT scion stem tissues away from the graft junction. The GFP observed near the graft junction is presumed to have spread via cell-to-cell diffusion, similar to the mode of siRNA translocation reported by Himber et al. (2003). Conversely, GFP detected at locations away from the graft junction is speculated to have spread through phloem-mediated long-distant transport, as discussed by Miyahara et al. (2024).

### Metabolic profiling of MT scion fruits harvested from transgrafted plant groups

Metabolomic analyses were conducted on five tomato fruits each from GFP-expressing transgrafted plant groups and their respective control grafted plant groups as listed in Table 1. PCA was performed based on the data matrix of liquid chromatography-mass spectrometry (LC-MS) data (positive ion mode, 61 ion peaks; negative ion mode, 38 ion peaks). The two-dimensional score plot graphs, combining PC1 and PC2, showed no separation of score plots between MT/MT and MTGFP/MT, Nb/MT and NbGFP/MT, and Nt/MT and NtGFP/MT for both data obtained in positive and negative ion modes (Supplementary Figure S12). These results indicated that the expression of GFP in the rootstock did not strongly influence the metabolic profiles of tomato fruits borne on MT scions of homo-transgraft (MTGFP/MT) or hetero-transgraft (NbGFP/MT and NtGFP/MT) groups. The ion peaks subjected to PCA were further analyzed using Student’s *t*-test. Two ion peaks in NbGFP/MT and one ion peak in NtGFP/MT exhibited significantly different abundances (P_FDR_<0.05) compared with their respective control grafted groups (Supplementary Table S6). Among them, ion peak ID P857 and N1415 showed lower abundances in NbGFP/MT compared with the control Nb/MT, while ion peak ID N366 showed higher abundances in NtGFP/MT compared with the control Nt/MT. To annotate ion peak ID N366 (retention time: 5.59 min; *m*/*z*: 593.30), tandem MS (MS/MS) analysis was conducted, and the resulting data were utilized for a mass spectral library search within the MassBank database (Horai et al. 2010). Kaempferol-3-*O*-rutinoside (MassBank Record: MSBNK-RIKEN-PR100665) was identified with the highest match score of 0.958. This compound has been previously reported in tomato fruits (Bovy et al. 2002; Le Gall et al. 2003). No significant difference was observed in the content of the toxic steroidal glycoalkaloid α-tomatine among the grafted plant lines (data not shown). These results indicated that the use of GFP-expressing rootstocks (tomato, *N. benthamiana*, or *N. tabacum*) did not influence the metabolic profiles of MT scion fruits.

Regarding Nb/MT and NbGFP/MT, transcriptome and proteome analyses were conducted on three tomato fruits harvested from MT tomato scions. The PCA score plot graphs revealed distinct transcriptomic profiles between Nb/MT and NbGFP/MT, while proteomic profiles showed no clear differences (Supplementary Figures S13, S14). There were 3409 DEGs between Nb/MT and NbGFP/MT, and 1548 DEGs were expressed more in the NbGFP/MT than in the Nb/MT. Additionally, there were 119 DEPs between the two groups NbGFP/MT and Nb/MT (Supplementary Table S4), with 57 DEPs highly expressed in the NbGFP/MT. GO terms significantly enriched in the DEGs and DEPs are shown in Supplementary Tables S3 and S5, respectively. These results suggest that using GFP-expressing *N. benthamiana* rootstocks may moderately affect the transcriptomic profiles and have a minor impact on the proteomic profiles of MT tomato scion fruits in NbGFP/MT plants.

## Conclusion

In this study, we investigated the translocation of transgene products from GM rootstocks to non-GM tomato scions in transgrafted plants. Initially, no critical differences were observed in the metabolite accumulation profiles of non-GM tomato scion fruits harvested from these transgrafted plants compared with respective control grafted plants (Supplementary Figure S12). However, similar to the movement of Luc protein in the homo-transgrafted tobacco plants (Miyahara et al. 2024), GFP was in fact detected in the stem of the non-GM tomato scions grafted onto the GFP-expressing tomato and GFP-expressing tobacco rootstocks (Table 2, Figure 3). Additionally, Luc was detected in the stems of hetero-transgrafted plants composed of non-GM tomato scions and the Luc-expressing *N. tabacum* rootstocks (Figure 1). In contrast, under our experimental conditions, neither GFP nor Luc was detected in the mature leaves and 10-DAB fruits (the edible part of tomato) of the non-GM tomato scion in any of the tested transgrafted plants (Table 2, Figure 1). These results suggest that the translocation of GFP and Luc from GM rootstocks to non-GM scions is limited to stem tissues in transgrafted plants. It should be noted that the transfer of GFP and Luc across the grafting unions was not consistently detected but occurred in some of the transgrafted plants. There are several potential reasons for the observed individual variations, such as different protein expression levels in the rootstock, the state of tissue fusion around the graft junction, and the condition of the individuals in response to environmental stresses. It is also possible that the amounts of translocated proteins in some plant samples were below the detection limit in our experiments. The factors underlying the individual variations in exogenous protein translocation and the sporadic accumulation of these proteins in stem tissues remain to be elucidated.

In conclusion, our results indicate that recombinant proteins, the translation products of foreign transgenes, could be transferred to non-GM portions in transgrafted plants in multiple cases. This raises concerns as to whether such non-GM edible portions of transgrafted plants can be uniformly treated as non-GM products. According to Japanese guidelines, points to be considered in safety assessments include potential health impacts on humans or livestock from new traits or alterations associated with the application of recombinant DNA technology. Therefore, if non-GM edible portions obtained through transgrafting contained recombinant proteins, it would be necessary to conduct individual assessments of the allergenicity or toxicity of the proteins according to the Food Sanitation Act and the Feed Safety Act in Japan.

## Abbreviations

DAB: days after breaker
MT: *Solanum lycopersicum* L. cv. Micro-Tom
MTGFP: transgenic *Solanum lycopersicum* L. cv. Micro-Tom expressing GFP
Nb: *Nicotiana benthamiana*
NbGFP: transgenic *Nicotiana benthamiana* expressing GFP
Nt: *Nicotiana tabacum* L. cv. SR1
NtGFP: transgenic *Nicotiana tabacum* L. cv. SR1 expressing GFP
NtLuc: transgenic *Nicotiana tabacum* L. cv. SR1 expressing Luc
RLU: relative light units

## Description of Supplementary Files

Figure S1. In planta Luc imaging in NtLuc/MT plants.

Figure S2. Semi-quantitative RT-PCR analysis of *Luc* gene transcript accumulation in MT scion stems of the NtLuc/MT plants.

Figure S3. Construction of the transgenic plants expressing GFP.

Figure S4. Confocal microscopy analysis of GFP accumulation in MT scion fruits of the MTGFP/MT, NbGFP/MT, and NtGFP/MT plants.

Figure S5. Confocal microscopy analysis of GFP accumulation in MT scion leaves of MTGFP/MT plants.

Figure S6. Confocal microscopy analysis of GFP accumulation in MT scion leaves of NbGFP/MT plants.

Figure S7. Confocal microscopy analysis of GFP accumulation in MT scion leaves of NtGFP/MT plants.

Figure S8. Confocal microscopy analysis of GFP accumulation in MT scion stems of MTGFP/MT plants.

Figure S9. Confocal microscopy analysis of GFP accumulation in MT scion stems of NbGFP/MT plants.

Figure S10. Confocal microscopy analysis of GFP accumulation in MT scion stems of NtGFP/MT plants.

Figure S11. Semi-quantitative RT-PCR analysis of *GFP* gene transcript accumulation in MT scion stems of the transgrafted plants.

Figure S12. PCA of metabolomic data of MT scion fruits from transgrafted plants.

Figure S13. PCA of transcriptomic data of MT scion fruits from NbGFP/MT and control Nt/MT plants.

Figure S14. PCA of proteomic data of MT scion fruits from NbGFP/MT and control Nt/MT plants.

Table S1. Primer pairs used for genotyping and RT-PCR.

Table S2. Alignment ratio for tomato and tobacco transcriptome data of each sample.

Table S3. Gene ontology analysis for the differentially expressed genes with increased expression in the NbGFP/MT group.

Table S4. The differential expression proteins list of the proteome analysis.

Table S5. Gene ontology analysis for the differential expression proteins with increased abundance ratio in the Nb/MT group.

Table S6. A list of ion peaks with statistically significant differences in abundance between transgrafted plant groups and their respective control grafted plant groups.
