# Supplementary material for: Translocation of green fluorescent protein in homo- and hetero-transgrafted plants
Source: Plant Biotechnol (Tokyo). 2024 Dec 25;41(4):345–56. doi: 10.5511/plantbiotechnology.24.0501b (PMC11897739; doi:10.5511/plantbiotechnology.24.0501b)
Supplement: Supplementary Data [file plantbiotechnology-41-4-24.0501b-s001.pdf]

Supplementary Table S1. Primer pairs used for genotyping and RT-PCR

Target	PCR type	Direction	DNA sequence (5' to 3')
GFP	genotyping, RT-PCR	Forward	CAAGGACGACGGCAACTACA
		Reverse	GACTGGGTGCTCAGGTAGTG
Luciferase	RT-PCR	Forward	ACACCCGAGGGGGATGATAA
		Reverse	GGCGACGTAATCCACGATCT
α-Tubulin (tomato)	RT-PCR	Forward	TGAACAACTCATAAGTGGCAAAG
		Reverse	TCCAGCAGAAGTGACCCAAGAC
Actin7 (tobacco)	RT-PCR	Forward	CCTCGCATCCCTTAGCACAT
		Reverse	AAAGGAAGATCTCGACCAAAGT
	genotyping	Forward	TGGGTGACGAAGCTCAATCC
		Reverse	AACCCTCGTAGATGGGGACA

Supplementary Table S2. Alignment ratio for tomato and tobacco transcriptome data of each sample

Sample	Reads	Alignment ratio for tomato transcript data (%)[a]	Alignment ratio for tobacco transcript data (%)[b]
Nb/MT1	15,236,572	75.32	18.49
Nb/MT2	20,249,074	75.86	17.69
Nb/MT3	16,451,204	75.38	18.44
NbGFP/MT1	16,490,440	77.60	18.45
NbGFP/MT2	15,350,948	76.74	17.54
NbGFP/MT3	18,854,786	77.53	18.54

[a]ITAG4.1_cDNA.fasta was used as tomato (*Solanum lycopersicum*) transcriptome data.
[b]Niben261_genome.annotation.transcripts.fasta was used as tobacco (*Nicotiana benthamiana*) transcriptome data.

Supplementary Table S3. Gene ontology analysis for the differential expression genes with increased expression in the NbGFP/MT group

Biological process	Gene ID	logFC: (NbGFP/MT) / (Nb/MT)	logCPM	PValue	FDR	Description
Lipid storage	Solyc08g078160.3	1.61	6.40	1.26.E-05	2.92.E-04	Oleosin
	Solyc02g086490.3	3.00	5.31	1.69.E-04	2.38.E-03	Oleosin
	Solyc03g119820.1	1.04	4.38	3.42.E-03	0.02	Oleosin
	Solyc12g010920.2	1.22	6.94	3.16.E-04	3.91.E-03	Oleosin
	Solyc06g034040.1	1.77	5.93	7.64.E-06	1.92.E-04	Oleosin
Cellular response to heat	Solyc03g007890.3	2.89	8.63	1.26.E-09	1.08.E-07	Histidine kinase_HSP90-like ATPase domain-containing protein
	Solyc12g015880.2	1.30	9.64	1.10.E-05	2.63.E-04	Molecular chaperone Hsp90-1
	Solyc06g036290.3	4.15	9.73	7.19.E-10	6.79.E-08	Histidine kinase_HSP90-like ATPase domain-containing protein
	Solyc03g115230.3	3.59	10.75	6.74.E-15	2.98.E-12	Clp R domain-containing protein
	Solyc03g097120.3	1.20	6.38	2.33.E-04	3.10.E-03	HSF-type DNA-binding domain-containing protein
	Solyc03g118340.3	0.95	8.93	2.13.E-03	0.02	Uncharacterized protein
	Solyc07g040680.3	1.66	6.29	4.74.E-06	1.30.E-04	HSF-type DNA-binding domain-containing protein

Only the biological process are shown that five or more genes in the differential expression genes were classified. The logFC means the log fold-change, the logCPM means the log counts per million.

Supplementary Table S4. The differential expression proteins list of the proteome analysis

Gene ID	Accession	Description	Abundance Ratio: (NbGFP/MT) / (Nb/MT)	PValue
Solyc06g062390.3	A0A3Q7GVG8	Stem 28 kDa glycoprotein	0.01	5.94E-16
Solyc10g049850.2	A0A3Q7JB80	TIP41-like protein	0.01	5.94E-16
Solyc07g045210.1	A0A3Q7HCV5	O-fucosyltransferase family protein	0.01	5.94E-16
Solyc11g069300.2	A0A3Q7IZP0	Protein kinase domain-containing protein	0.01	5.94E-16
Solyc01g006110.3	A0A3Q7E7S5	PP2A regulatory subunit TAP46	0.01	5.94E-16
Solyc11g065930.2	A0A3Q7IYN3	FAD-binding PCMH-type domain-containing protein	0.01	5.94E-16
Solyc09g014530.3	A0A3Q7HYH9	Bet_v_1 domain-containing protein	0.02	5.94E-16
Solyc09g007160.3	A0A3Q7HVQ8	Uncharacterized protein	0.07	4.98E-14
Solyc09g005400.3	A0A3Q7HXJ8	Bet_v_1 domain-containing protein	0.07	5.94E-16
Solyc06g071530.3	A0A3Q7H1I0	60S ribosomal protein L44	0.14	4.46E-09
Solyc04g080540.2	K4BVD3	ATP synthase subunit d, mitochondrial	0.20	1.28E-07
Solyc01g105410.3	A0A3Q7FB87	Protein EXORDIUM-like 2	0.29	4.29E-09
Solyc03g020060.3	I3QHF0	Proteinase inhibitor II	0.32	8.33E-09
Solyc09g015000.3	A0A3Q7HZL2	SHSP domain-containing protein	0.33	6.09E-09
Solyc01g102960.3	A0A3Q7F9P0	SHSP domain-containing protein	0.33	7.99E-06
Solyc01g080010.2	A0A3Q7EHP2	Peptidase A1 domain-containing protein	0.33	2.29E-07
Solyc05g051260.3	A0A3Q7GKY0	GH10 domain-containing protein	0.34	1.80E-02
Solyc09g090390.2	A0A3Q7I7G5	Glycine-rich cell wall structural protein-like	0.35	1.20E-03
Solyc07g064210.2	K4CHA2	AAI domain-containing protein	0.35	5.40E-07
Solyc06g082800.3	A0A3Q7HY14	Mitochondrial import receptor subunit TOM40-1-like	0.35	4.22E-02
Solyc02g062770.2	A0A3Q7EYV7	Uncharacterized protein	0.35	3.51E-04
Solyc01g107670.2	A0A3Q7ESD4	LRRNT_2 domain-containing protein	0.36	1.98E-03
Solyc07g007760.3	A0A3Q7H3Y0	Knot1 domain-containing protein	0.36	3.35E-06
Solyc08g068150.4	B2ZPK7	BURP domain-containing protein	0.36	1.07E-06
Solyc05g053350.3	A0A3Q7GLX5	Desiccation-related protein PCC13-62-like	0.37	1.72E-04
Solyc10g007260.3	K4CXQ6	UBC13-2	0.38	9.13E-03
Solyc06g062690.3	A0A3Q7GY01	Nucleosome assembly protein 1	0.38	2.99E-02
Solyc09g097850.1	A0A3Q7I8T9	Cysteine proteinase inhibitor	0.38	2.77E-06
Solyc02g089920.2	A0A3Q7FXS7	LysM domain receptor-like kinase 4	0.39	8.30E-03
Solyc08g067290.3	A0A3Q7IKK8	RING-type domain-containing protein	0.39	3.73E-02
Solyc03g083440.3	A0A3Q7FKX4	Glutamate synthase (NADH)	0.40	4.10E-02
Solyc02g071700.3	A0A3Q7F2J4	Triacylglycerol lipase	0.41	1.48E-03
Solyc01g104380.3	Q9M509	Plantacyanin	0.41	1.29E-03
Solyc02g078920.3	A0A3Q7F4A9	Aspergillus nuclease S(1)	0.42	7.53E-03
Solyc02g070510.3	A0A3Q7F2F3	Proteasome subunit alpha type	0.42	2.22E-03
Solyc11g020330.1	A0A3Q7ITP5	SHSP domain-containing protein	0.42	5.51E-03
Solyc11g068895.1	A0A3Q7IZG1	LEA_2 domain-containing protein	0.42	2.84E-02
Solyc07g049135.1	P14903	Fruit-specific protein	0.43	2.91E-04
Solyc03g113930.2	A0A3Q7FR32	SHSP domain-containing protein	0.43	7.41E-05
Solyc03g118690.3	A0A3Q7FSL1	TPR_REGION domain-containing protein	0.43	3.06E-02
Solyc09g092170.2	A0A3Q7IA20	Beta-galactosidase	0.43	2.58E-03
Solyc03g082420.3	Q95661	Small heat shock protein, chloroplastic	0.44	9.51E-05
Solyc10g005560.3	A0A3Q7I9N5	Ubiquitin-like domain-containing protein	0.44	1.70E-04
Solyc07g007750.3	B1N678	Defensin protein	0.44	5.75E-04
Solyc12g010960.2	A0A3Q7J4Q6	PKS_ER domain-containing protein	0.46	5.45E-04
Solyc09g007010.1	Q0H8U4	Pathogenesis-related protein	0.46	4.25E-04
Solyc04g074450.2	A0A3Q7G4Y3	Protein EXORDIUM-like 2	0.49	1.39E-02
Solyc12g011310.2	A0A3Q7J4K1	Glutathione transferase	0.49	2.15E-03
Solyc07g066030.3	A0A3Q7HIN5	Proteasome activator subunit 4	0.49	4.10E-03
Solyc02g064940.1	A0A3Q7FKJ0	LRRNT_2 domain-containing protein	0.49	2.79E-03
Solyc05g009940.3	A0A3Q7GDT2	Cytochrome b561 domain-containing protein	0.49	2.99E-02
Solyc04g078540.3	A0A3Q7G8Y1	Low-temperature-induced cysteine proteinase-like	0.52	1.71E-02
Solyc04g009630.3	A0A3Q7G077	Alpha-glucosidase	0.52	9.09E-03
Solyc07g005960.3	A0A3Q7H2W6	Carboxypeptidase	0.53	2.17E-02
Solyc06g008260.3	A0A3Q7GMW4	Ribosomal_L14e domain-containing protein	0.54	1.68E-02
Solyc06g076570.2	A0A3Q7H3Z8	SHSP domain-containing protein	0.55	3.52E-02
Solyc02g079170.3	A0A3Q7F4F8	NADH:ubiquinone reductase (non-electrogenic)	0.55	3.50E-02

Solyc11g069270.2	Q9LLT0	Beta-galactosidase	0.56	2.97E-02
Solyc08g081730.3	A0A3Q7HWG7	Reticulon-like protein	0.56	4.35E-02
Solyc01g094790.3	A0A3Q7F5F8	Cysteine synthase	0.57	4.99E-02
Solyc03g007810.3	A0A3Q7G2Q9	Pyruvate kinase	0.58	4.64E-02
Solyc07g006560.3	A0A3Q7H3Z1	Ribonuclease T(2)	0.58	4.67E-02
Solyc12g056220.2	K4DFV3	Plasmamembrane intrinsic protein 13	1.62	3.14E-02
Solyc10g079860.2	Q43778	Glucan endo-1,3-beta-D-glucosidase	1.64	2.36E-02
Solyc10g055800.2	A0A3Q7IHS3	Chitin-binding type-1 domain-containing protein	1.64	4.97E-02
Solyc03g122360.3	A0A3Q7FUI5	Cytochrome P450 71A1-like	1.66	2.99E-02
Solyc01g087540.3	A0A3Q7F2V6	Uncharacterized protein	1.66	2.35E-02
Solyc10g082030.2	A0A3Q7ILV3	Thioredoxin-dependent peroxiredoxin	1.68	2.67E-02
Solyc03g046380.1	A0A3Q7FHA2	Seed maturation protein	1.68	3.67E-02
Solyc01g088400.3	A0A3Q7EJF1	Protein ECERIFERUM 1-like	1.70	3.06E-02
Solyc02g065250.2	A0A3Q7EZJ3	Salicylic acid-binding protein	1.72	2.57E-02
Solyc07g042440.3	A0A3Q7H8S0	Thioredoxin-dependent peroxiredoxin	1.72	1.12E-02
Solyc02g092670.2	A0A3Q7FB69	Subtilisin-like protease SBT1.7	1.74	1.61E-02
Solyc06g060340.3	P54773	Photosystem II 22 kDa protein, chloroplastic	1.75	1.19E-02
Solyc02g067750.4	B1VK36	Carbonic anhydrase	1.77	1.53E-02
Solyc08g080490.3	A0A3Q7HTE7	AAI domain-containing protein	1.78	1.46E-02
Solyc07g065110.1	A0A3Q7HI57	AAI domain-containing protein	1.78	1.80E-02
Solyc09g056385.1	A0A3Q7I3M8	DOMON domain-containing protein	1.89	1.66E-02
Solyc08g066240.3	A0A3Q7IJX7	Histidine decarboxylase	1.91	3.83E-03
Solyc09g014750.1	A0A3Q7IVC8	Em protein H2	1.94	2.87E-03
Solyc08g079090.3	A0A3Q7HV28	Monocopper oxidase-like protein SKU5	1.97	1.13E-02
Solyc09g010210.3	Q42872	Endoglucanase	2.00	1.99E-03
Solyc01g060020.3	A0A3Q7EEZ8	(1->3)-beta-glucan endohydrolase	2.13	6.38E-03
Solyc10g074740.1	A0A3Q7IJ47	Calcium-binding protein CML42	2.14	6.23E-03
Solyc02g036350.3	A4ZYQ6	1-aminocyclopropane-1-carboxylate oxidase	2.17	1.26E-02
Solyc06g074710.1	A0A3Q7H2Z1	Agmatine coumaroyltransferase-2-like	2.17	3.81E-03
Solyc01g106620.2	B2LW68	PR1 protein	2.27	2.88E-02
Solyc02g077040.4	Q156I2	Phytophthora-inhibited protease 1	2.28	1.38E-05
Solyc11g073250.2	A0A3Q7J176	Histone H2A	2.29	2.77E-03
Solyc01g111350.3	A0A3Q7EUC4	Nodulin-like domain-containing protein	2.31	1.13E-02
Solyc01g091530.3	A0A3Q7ELA6	FAS1 domain-containing protein	2.32	3.48E-06
Solyc09g007910.3	A0A3Q7HWL4	Phenylalanine ammonia-lyase	2.39	6.16E-03
Solyc02g079950.3	Q672Q6	Photosystem II oxygen-evolving complex protein 3	2.41	2.42E-02
Solyc03g119360.3	A0A3Q7FW72	40S ribosomal protein S7	2.41	2.17E-02
Solyc01g066860.3	A0A3Q7EZE4	Protein-serine/threonine phosphatase	2.44	3.51E-04
Solyc01g081570.3	A0A3Q7EIW9	Carboxyl-terminal peptidase	2.47	1.90E-02
Solyc04g082010.1	P17340	Plastocyanin, chloroplastic	2.48	2.64E-05
Solyc03g111690.3	A0A3Q7FPN1	Pectate lyase	2.51	2.24E-06
Solyc02g094440.3	A0A3Q7G0J8	Translocon-associated protein subunit beta	2.56	1.45E-02
Solyc01g111970.3	A0A3Q7FF59	L-ascorbate oxidase homolog	2.62	1.49E-06
Solyc02g069490.3	A0A0C6G3Q8	Sterol side chain reductase	2.65	1.56E-02
Solyc11g066290.2	A0A3Q7IZK1	Metallophos domain-containing protein	2.66	5.78E-05
Solyc04g076780.3	A0A3Q7G5L3	Peroxidase	2.68	2.79E-05
Solyc06g073760.3	A0A3Q7HVR3	Lysosomal beta glucosidase-like	2.70	6.26E-03
Solyc12g055730.2	A0A3Q7JBG6	Lipase_3 domain-containing protein	2.76	2.90E-07
Solyc05g054090.3	A0A3Q7HGQ3	Fibrous sheath CABYR-binding protein-like	2.76	2.85E-07
Solyc10g078770.2	Q00747	Protein LE25	2.80	1.96E-07
Solyc10g055810.2	Q05538	Basic 30 kDa endochitinase	3.12	5.47E-10
Solyc02g084850.3	P22240	Abscisic acid and environmental stress-inducible protein TAS14	3.16	6.23E-06
Solyc11g006910.2	A0A3Q7IPC7	Ferredoxin	3.46	1.18E-03
Solyc10g080840.1	A0A3Q7ILQ8	Cytochrome P450	3.52	1.05E-10
Solyc03g083910.5	A0A220QMI6	Beta-fructofuranosidase	3.59	2.27E-11
Solyc03g020040.3	A0A3Q7FEJ3	Proteinase inhibitor type-2	4.10	1.90E-05
Solyc03g019690.1	A0A3Q7FGU5	Proteinase inhibitor I3, Kunitz legume	4.14	5.76E-10
Solyc02g071030.2	P07370	Chlorophyll a-b binding protein 1B, chloroplastic	4.43	4.73E-08
Solyc02g077710.1	A0A3Q7F467	Protein E6	4.85	8.43E-08
Solyc01g006230.3	A0A3Q7E812	Cysteine protease	7.71	5.35E-07

Solyc04g080620.3	A0A3Q7GB08	Mannan endo-1,4-beta-mannosidase	100.00	5.94E-16
Solyc12g035240.2	A0A3Q7J7C5	DUF1421 domain-containing protein	100.00	5.94E-16

Supplementary Table S5. Gene ontology analysis for the differential expression proteins with increased abundance ratio in the Nb/MT group

Biological process	Gene ID	Accession	Description	Abundance Ratio: (NbGFP/MT) / (Nb/MT)	PValue
Protein complex oligomerization	Solyc01g102960.3	A0A3Q7F9P0	SHSP domain-containing protein	0.33	7.99E-06
	Solyc03g113930.2	A0A3Q7FR32	SHSP domain-containing protein	0.43	7.41E-05
	Solyc09g015000.3	A0A3Q7HZL2	SHSP domain-containing protein	0.33	6.09E-09
	Solyc11g020330.1	A0A3Q7ITP5	SHSP domain-containing protein	0.42	5.51E-03
	Solyc06g076570.2	A0A3Q7H3Z8	SHSP domain-containing protein	0.55	3.52E-02

Only the biological process are shown that five or more proteins in the differential expression proteins were classified.

Supplementary Table S6. A list of ion peaks with statistically significant differences in abundance between transgrafted plant groups and their respective control grafted plant groups

Ion peak ID	Retention time (min)	*m/z*	Combinations	Fold-difference (transgrafted plant group / control)	p.ajusted (FDR)	Description
P857	4.16	205.10	NbGFP/MT vs Nb/MT	0.39	0.01306	low in NbGFP/MT
N1415	5.12	431.18	NbGFP/MT vs Nb/MT	0.64	0.00601	low in NbGFP/MT
N366	5.60	593.30	NtGFP/MT vs Nt/MT	2.49	0.00033	high in NtGFP/MT
