# Supplementary material for: Translocation of green fluorescent protein in homo- and hetero-transgrafted plants
Source: Plant Biotechnol (Tokyo). 2024 Dec 25;41(4):345–56. doi: 10.5511/plantbiotechnology.24.0501b (PMC11897739; doi:10.5511/plantbiotechnology.24.0501b)
Supplement: Supplementary Data [file plantbiotechnology-41-4-24.0501b-s002.pdf]

Supplementary Figures S1–S14

Translocation of green fluorescent protein in homo- and hetero-transgrafted plants

Takumi Ogawa, Kanae Kato, Harue Asuka, Yumi Sugioka, Tomofumi Mochizuki, Hirokazu Fukuda, Takumi Nishiuchi, Taira Miyahara, Hiroaki Kodama and Daisaku Ohta

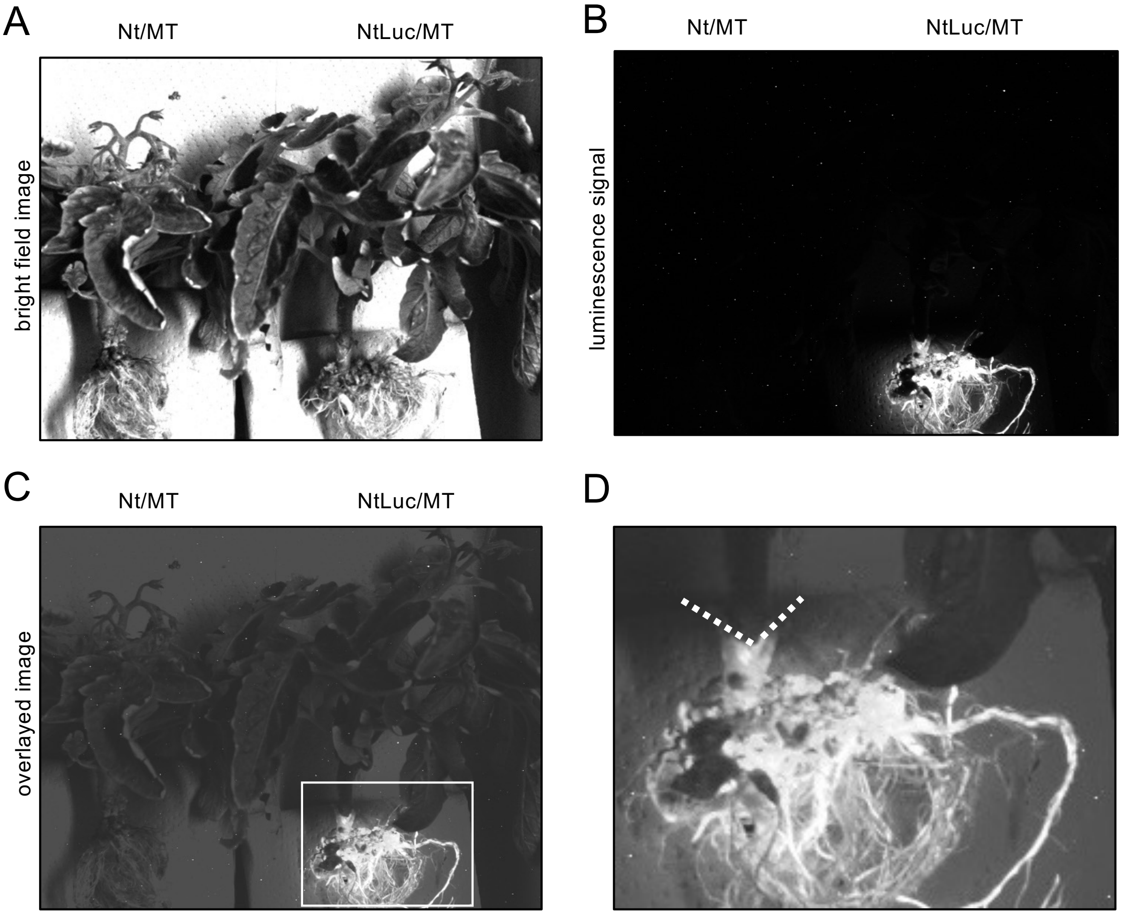

Supplementary Figure S1. *In planta* Luc imaging in NtLuc/MT plants. A bright image (A), a luminescence signal image (B), an overlayed image of A and B (C), and a magnified image from the inset of C (D) were shown. White dashed line indicate the junction between scion and rootstock.

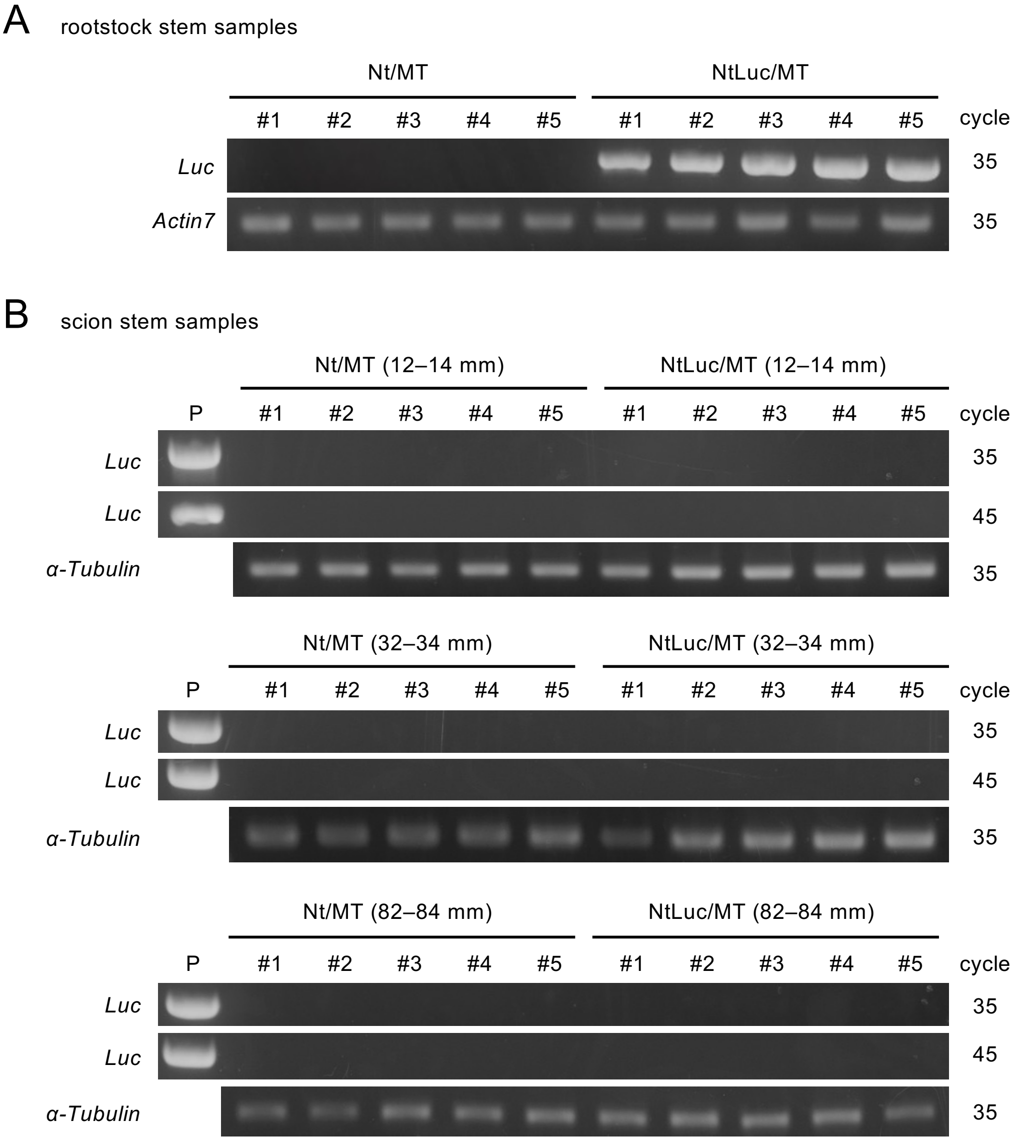

Supplementary Figure S2. Semi-quantitative RT-PCR analysis of *Luc* gene transcript accumulation in MT scion stems of the NtLuc/MT plants. Results obtained from (A) rootstock and (B) scion samples are shown. In lanes P, we loaded amplification products of the positive control samples (rootstock stem portions of NtLuc/MT #1). The number of PCR cycles is indicated on the right side of the gel images. The tomato *α-Tubulin* gene (NCBI number: LOC101254013) and the predicted *N. tabacum Actin7* gene (NCBI number: LOC107831145) was used as reference genes for the RT-PCR analysis.

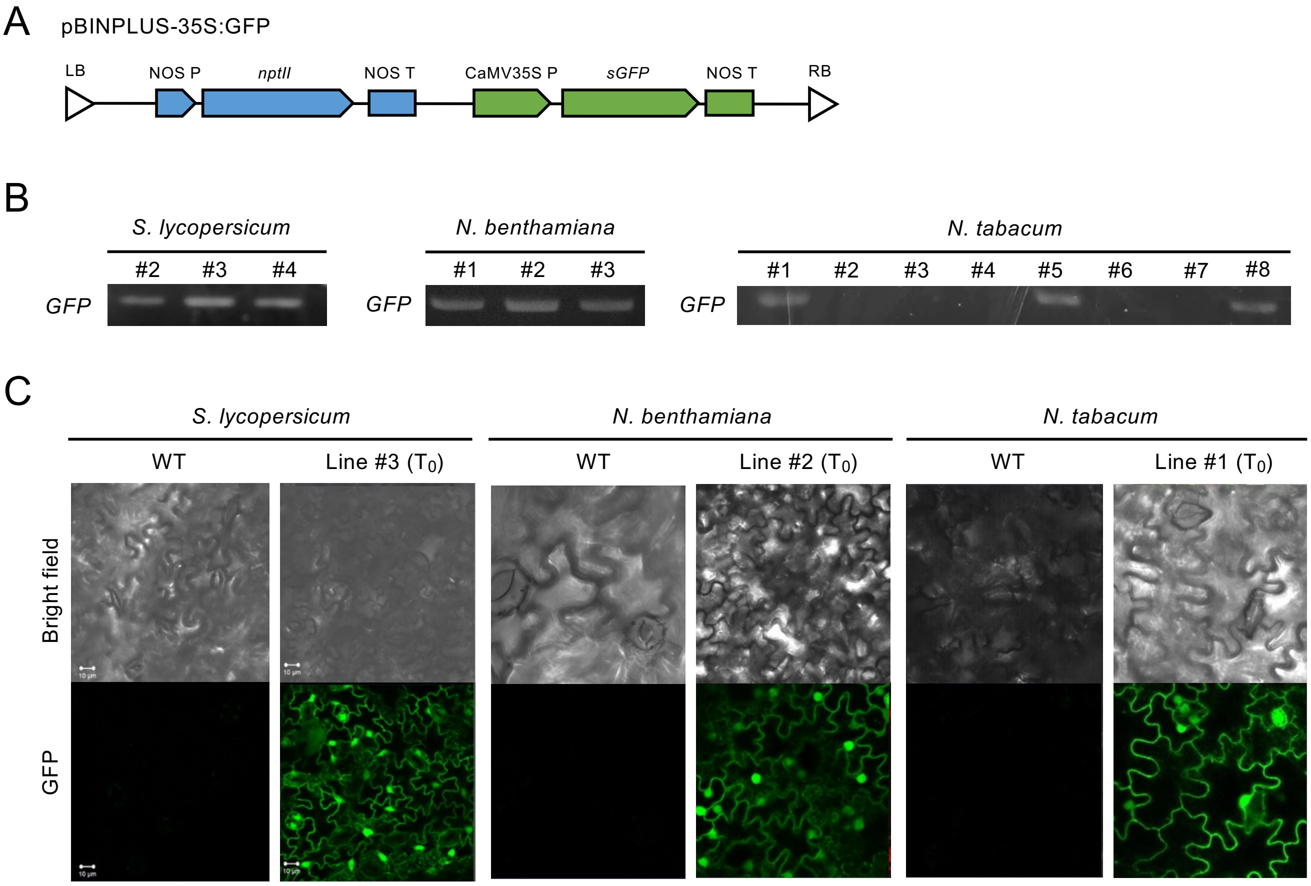

Supplementary Figure S3. Construction of the transgenic plants expressing GFP. (A) Schematic representation of the T-DNA region in the plasmid vector pBINPLUS-35S:GFP used for agrobacterium-mediated plant transformation. The T-DNA region includes LB (the left border sequence of T-DNA), NOS P (nopaline synthase promoter), *nptII* (neomycin-kanamycin phosphotransferase II gene), NOS T (nopaline synthase gene terminator), CaMV35S P (Cauliflower mosaic virus 35S promoter), sGFP (sGFP gene), and RB (the right border sequence of T-DNA). (B) Genotyping of T_0 regenerated plants. PCR were performed using genomic DNA as a template and a set of GFP specific primers. (C) Confocal microscopy analysis of GFP expression in the leaf epidermal cells of T_0 regenerated plants. Images were captured using the LSM700 system with a 488 nm laser for excitation and an emission filter setting of 490–520 nm.

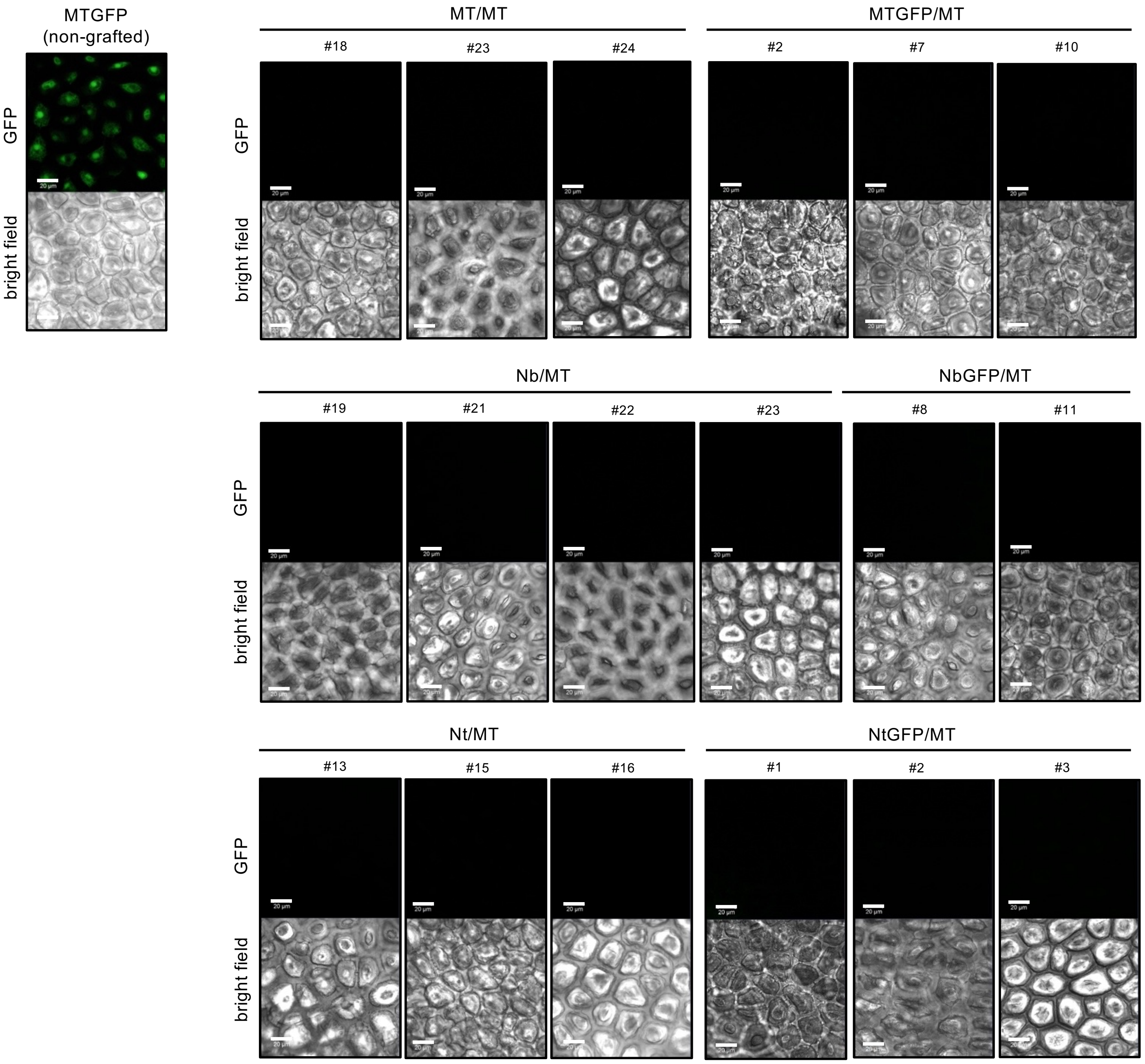

Supplementary Figure S4. Confocal microscopy analysis of GFP accumulation in MT scion fruits of MTGFP/MT, NbGFP/MT, and NtGFP/MT plants. Pericarp tissues from 10 DAB fruits were used for the observation. Images were captured using the LSM700 system with a 488 nm laser for excitation and an emission filter setting of 490–520 nm. Representative images are shown. A non-grafted MTGFP plant was used as a positive control for GFP fluorescence detection (shown in the top left panel).

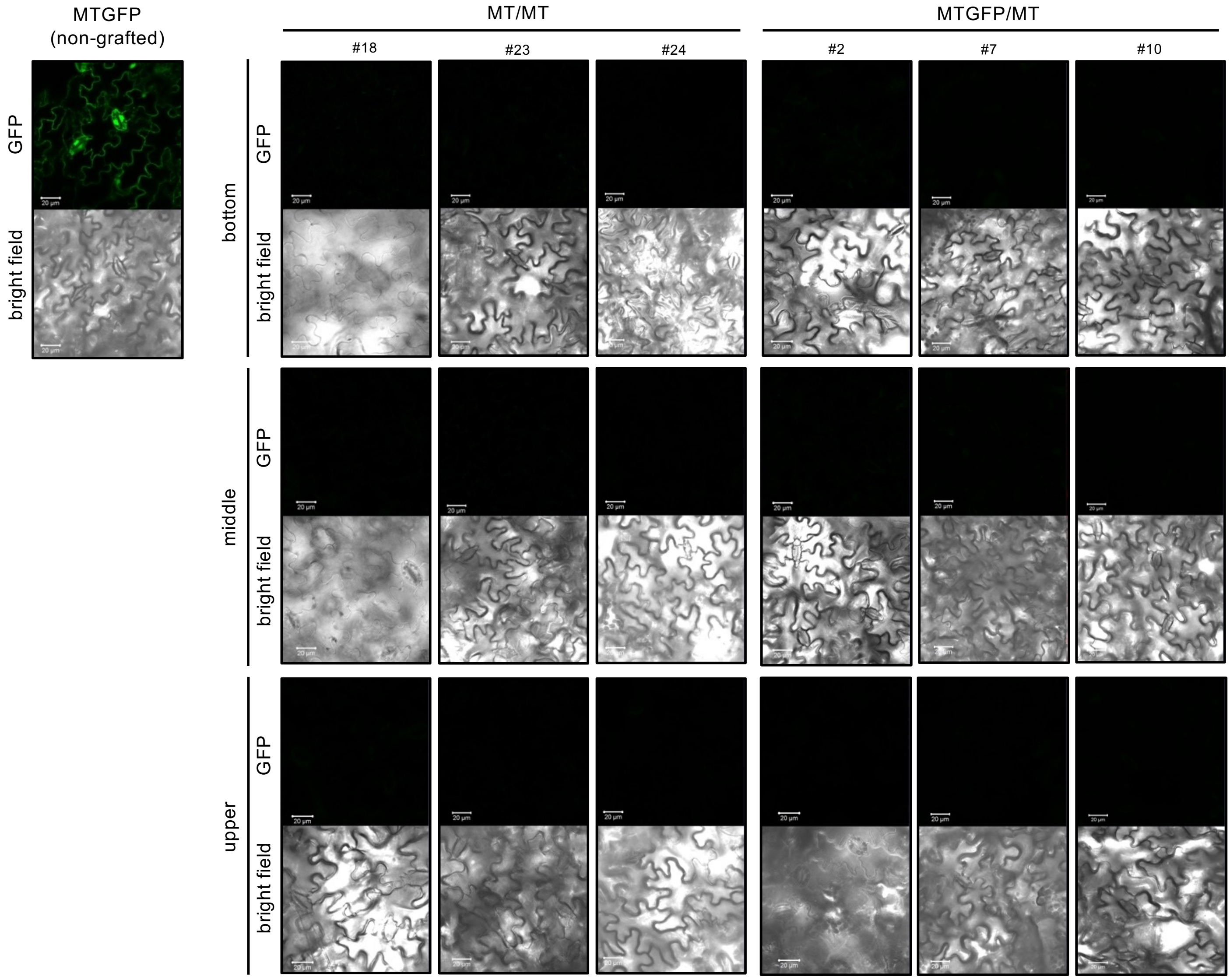

Supplementary Figure S5. Confocal microscopy analysis of GFP accumulation in MT scion leaves of MTGFP/MT plants. Leaves from the bottom (1st leaf counted from the graft junction), middle (4th–6th leaf counted from the graft junction), and upper (7th–12th leaf counted from the graft junction) parts of the grafted plants were used for the observation. Images were captured using the LSM700 system with a 488 nm laser for excitation and an emission filter setting of 490–520 nm. Representative images are shown. A non-grafted MTGFP plant was used as a positive control for detecting GFP fluorescence (shown in the top left panel).

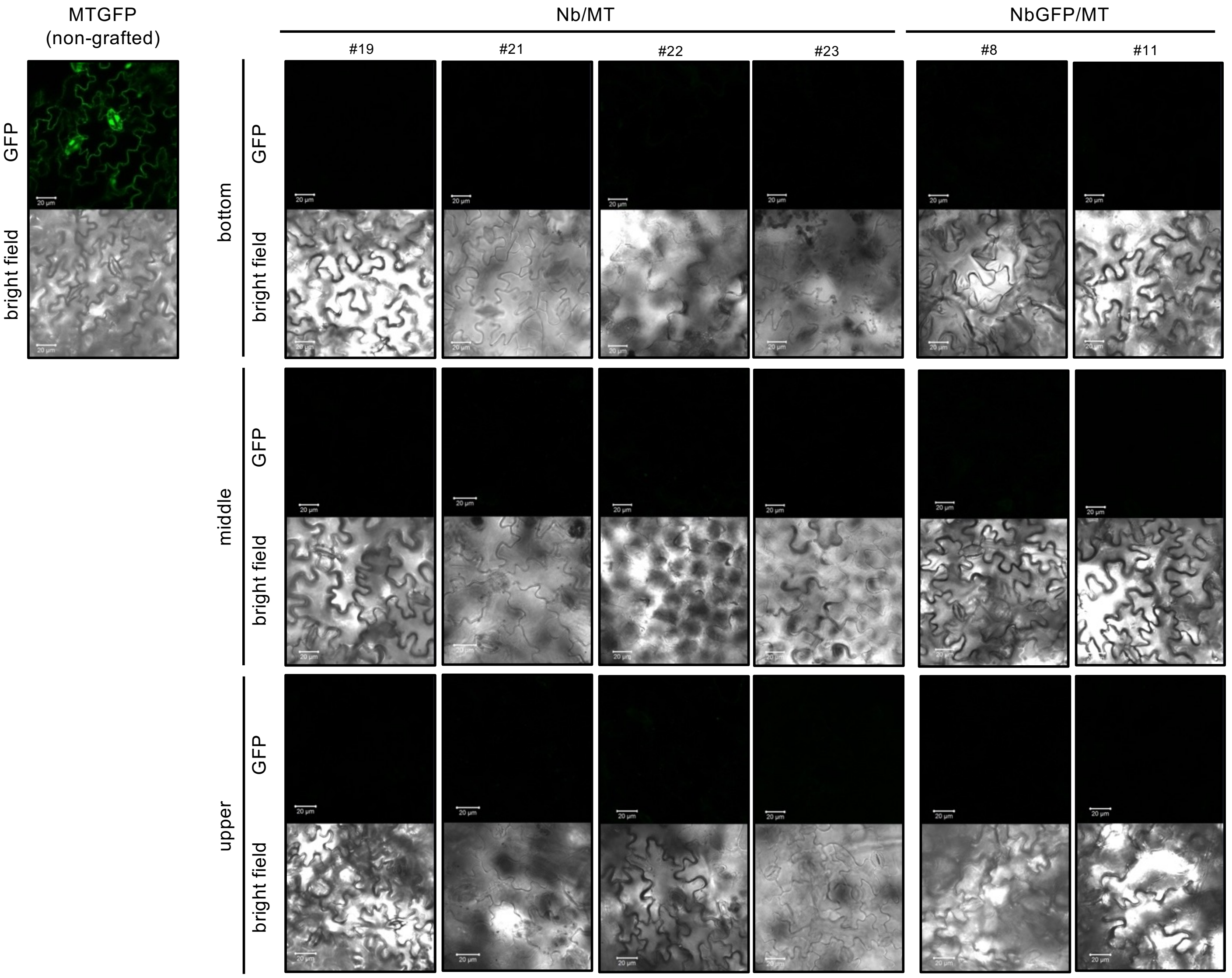

Supplementary Figure S6. Confocal microscopy analysis of GFP accumulation in MT scion leaves of NbGFP/MT plants. Leaves from the bottom (1st leaf counted from the graft junction), middle (4th–6th leaf counted from the graft junction), and upper (7th–12th leaf counted from the graft junction) parts of the grafted plants were used for the observation. Images were captured using the LSM700 system with a 488 nm laser for excitation and an emission filter setting of 490–520 nm. Representative images are shown. A non-grafted MTGFP plant was used as a positive control for detecting GFP fluorescence (shown in the top left panel).

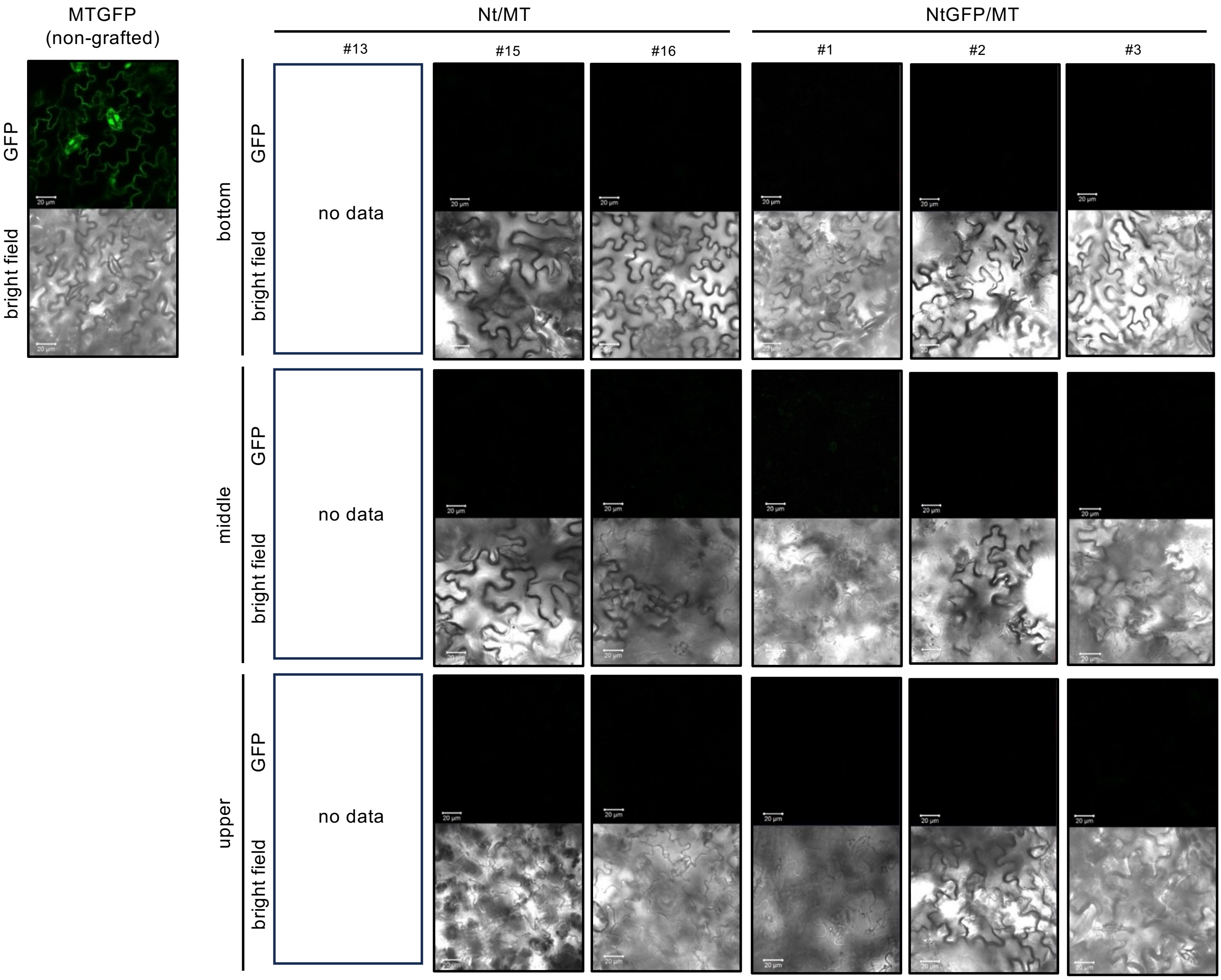

Supplementary Figure S7. Confocal microscopy analysis of GFP accumulation in MT scion leaves of NtGFP/MT plants. Leaves from the bottom (1st leaf counted from the graft junction), middle (4th–6th leaf counted from the graft junction), and upper (7th–12th leaf counted from the graft junction) parts of the grafted plants were used for the observation. Images were captured using the LSM700 system with a 488 nm laser for excitation and an emission filter setting of 490–520 nm. Representative images are shown. A non-grafted MTGFP plant was used as a positive control for detecting GFP fluorescence (shown in the top left panel). The data from a Nt/MT plant (#13) are missing due to an error in saving photo data.

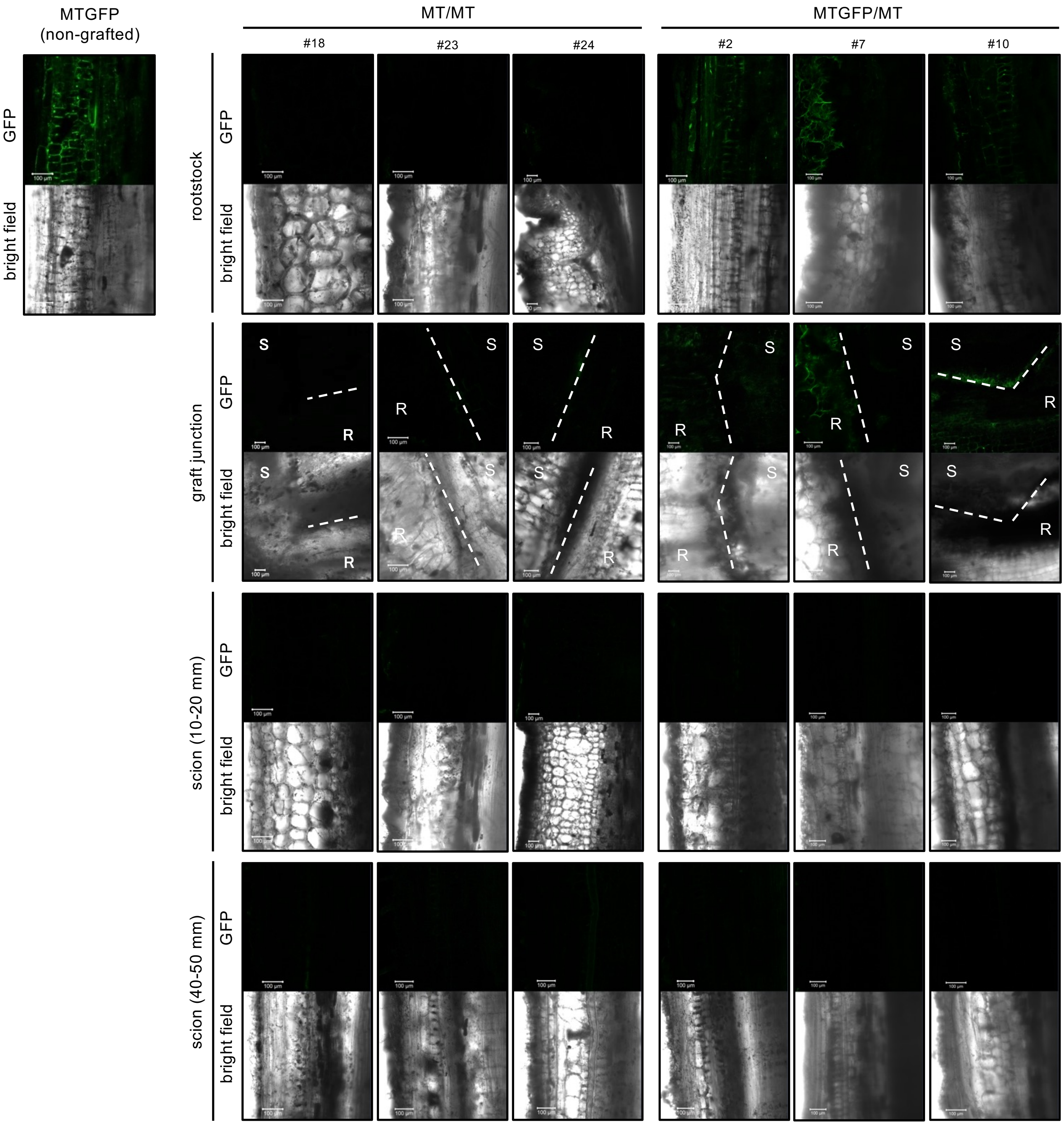

Supplementary Figure S8. Confocal microscopy analysis of GFP accumulation in MT scion stems of MTGFP/MT plants. Stem sections from the rootstock, graft junction, and scion portions (10–20 mm and 40–50 mm away from the graft junction) of the grafted plants were used for the observation. Images were captured using the LSM700 system with a 488 nm laser for excitation and an emission filter setting of 490–520 nm. Representative images are shown. A non-grafted MTGFP was used as the positive control for GFP fluorescence detection (shown in the top left panel). S: scion, R: rootstock. White dashed line indicate the junction between scion and rootstock.

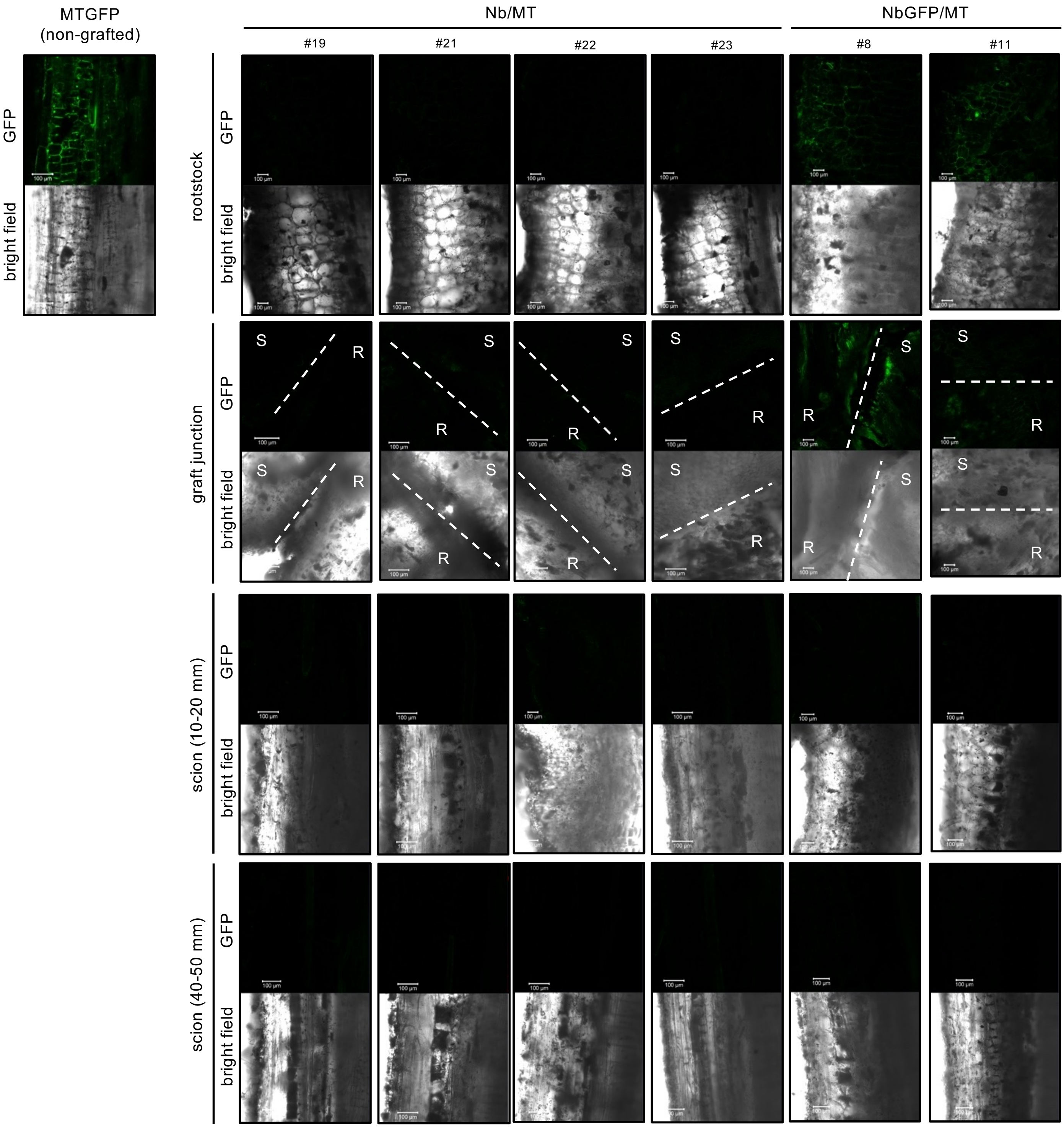

Supplementary Figure S9. Confocal microscopy analysis of GFP accumulation in MT scion stems of NbGFP/MT plants. Stem sections from the rootstock, graft junction, and scion portions (10–20 mm and 40–50 mm away from the graft junction) of the grafted plants were used for the observation. Images were captured using the LSM700 system with a 488 nm laser for excitation and an emission filter setting of 490–520 nm. Representative images are shown. A non-grafted MTGFP was used as the positive control for GFP fluorescence detection (shown in the top left panel). S: scion, R: rootstock. White dashed line indicate the junction between scion and rootstock.

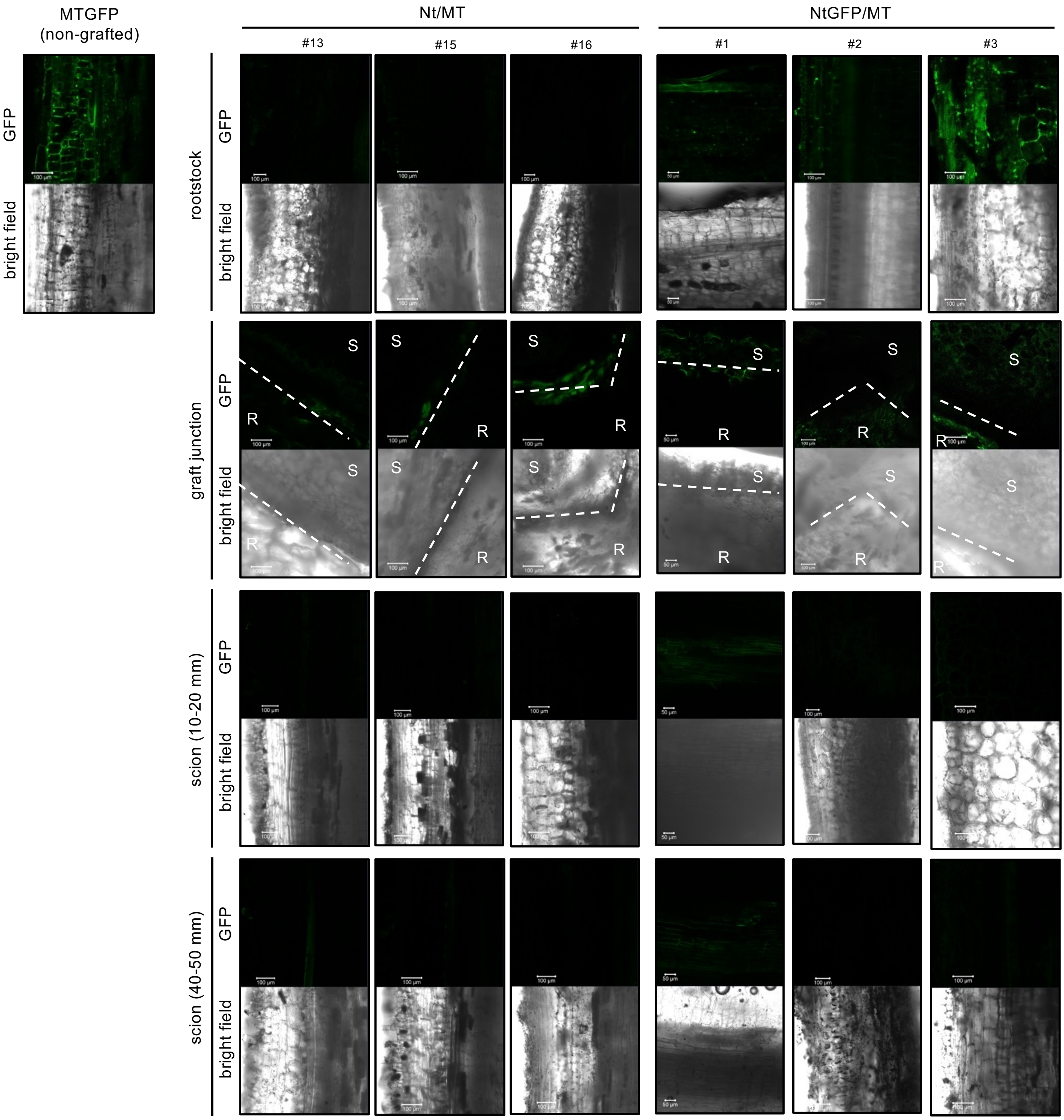

Supplementary Figure S10. Confocal microscopy analysis of GFP accumulation in MT scion stems of NtGFP/MT plants. Stem sections from the rootstock, graft junction, and scion portions (10–20 mm and 40–50 mm away from the graft junction) of the grafted plants were used for the observation. Images were captured using the LSM700 system with a 488 nm laser for excitation and an emission filter setting of 490–520 nm. Representative images are shown. A non-grafted MTGFP was used as the positive control for GFP fluorescence detection (shown in the top left panel). S: scion, R: rootstock. White dashed line indicate the junction between scion and rootstock.

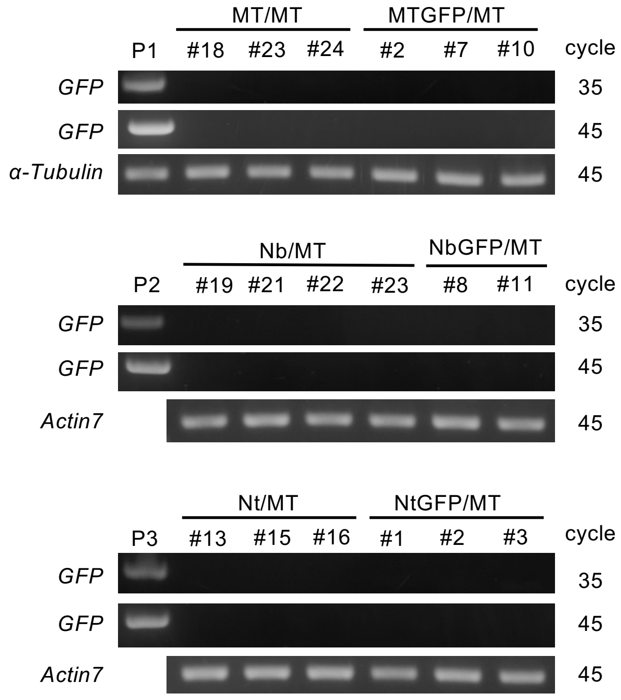

Supplementary Figure S11. Semi-quantitative RT-PCR analysis of *GFP* gene transcript accumulation in MT scion stem portions of the transgrafted plants. All stem samples were prepared from positions 10–20 mm away from the graft junction. In lanes P1, P2, and P3, we loaded amplification products of positive control samples from the rootstock stem portions of MTGFP/MT #10, NbGFP/MT #8, and NtGFP/MT #1, respectively. The number of PCR cycles is indicated on the right side of the gel images. The tomato *α-Tubulin* gene (NCBI number: LOC101254013) and the predicted *N. tabacum Actin7* gene (NCBI number: LOC107831145) was used as reference genes for the RT-PCR analysis.

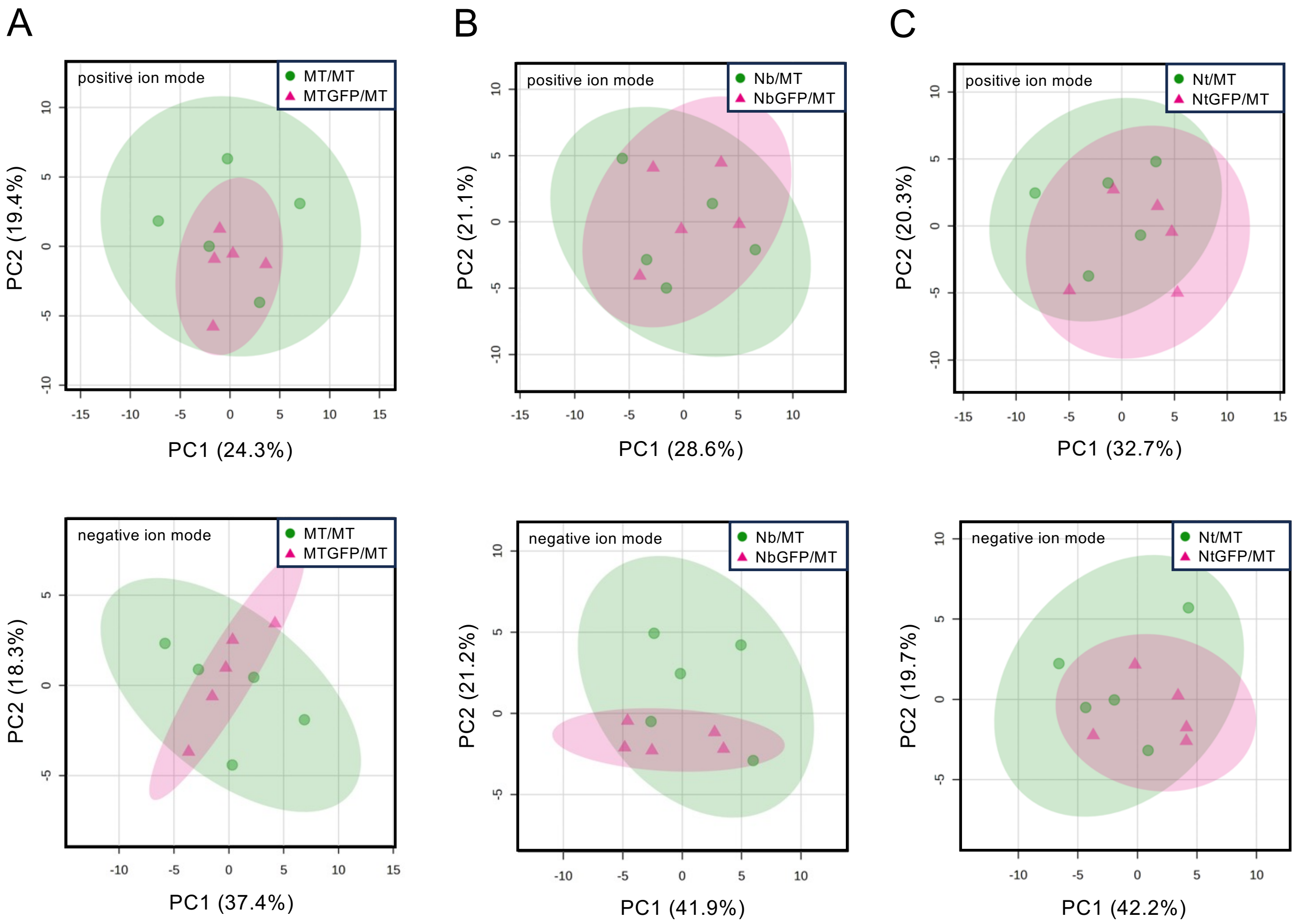

Supplementary Figure S12. PCA of metabolomic data of MT scion fruits from transgrafted plants. (A) MT/MT vs. MTGFP/MT, (B) Nb/MT vs. NbGFP/MT, (C) Nt/MT vs. NtGFP/MT. The two-dimensional score plot graph composed of the combination of PC1 and PC2 is shown. The upper and lower panels represented the score plot graphs for data obtained in positive and negative ion modes, respectively. Each plot represents an individual sample (n = 5). Percentage values in parentheses are the respective contribution ratios. The 95% confidence regions are shown by ovals, filled in green (MT/MT, Nb/MT, and Nt/MT) or magenta (MTGFP/MT, NbGFP/MT, and NtGFP/MT).

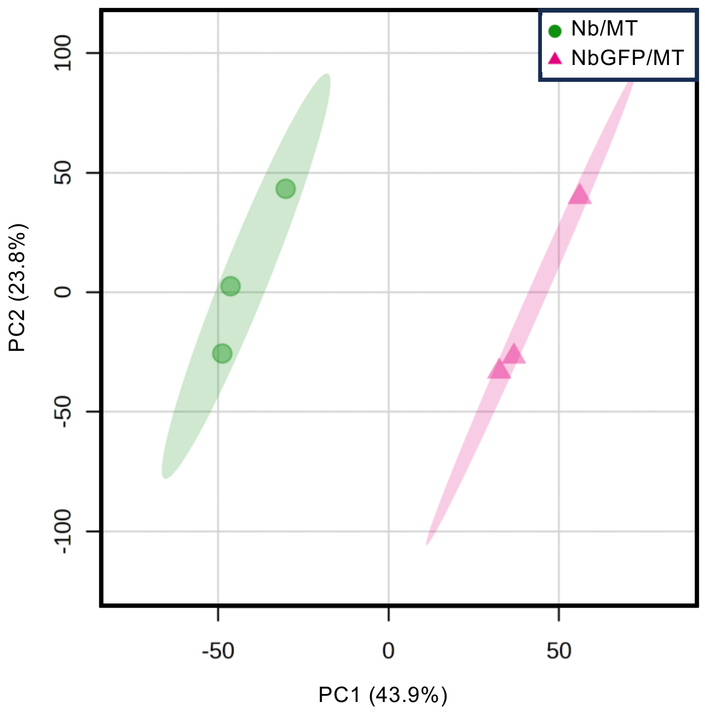

Supplementary Figure S13. PCA of transcriptomic data of MT scion fruits from NbGFP/MT and control Nt/MT plants. The two-dimensional score plot graph composed of the combination of PC1 and PC2 is shown. Each plot represents an individual sample (n = 3). Percentage values in parentheses are the respective contribution ratios. The 95% confidence regions are shown by ovals, filled in green (Nb/MT) or magenta (NbGFP/MT).

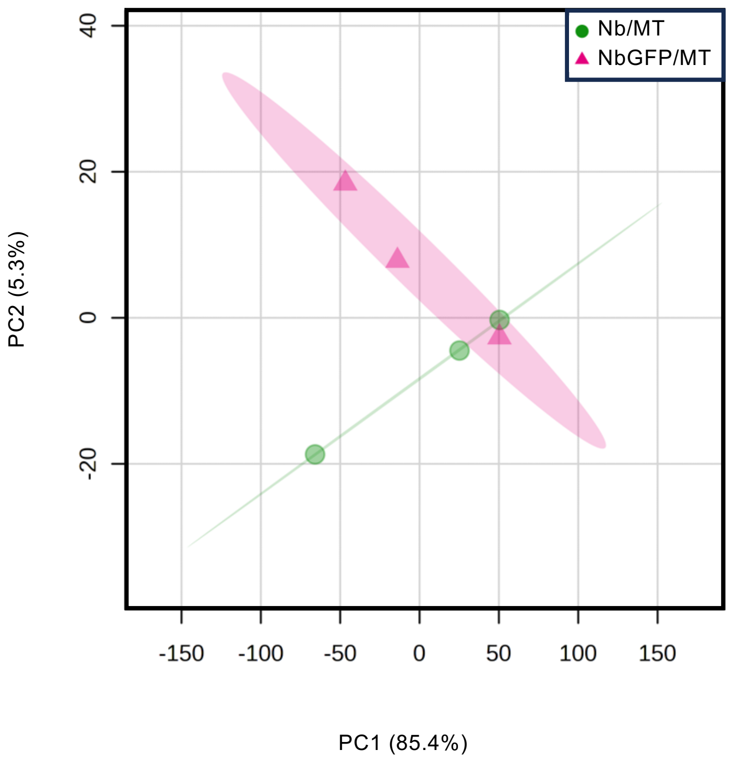

Supplementary Figure S14. PCA of proteomic data of MT scion fruits from NbGFP/MT and control Nt/MT plants. The two-dimensional score plot graph composed of the combination of PC1 and PC2 is shown. Each plot represents an individual sample (n = 3). Percentage values in parentheses are the respective contribution ratios. The 95% confidence regions are shown by ovals, filled in green (Nb/MT) or magenta (NbGFP/MT).
